# CXCL9/10-engineered dendritic cells promote T cell activation and enhance immune checkpoint blockade for lung cancer

**DOI:** 10.1016/j.xcrm.2024.101479

**Published:** 2024-03-21

**Authors:** Raymond J. Lim, Ramin Salehi-Rad, Linh M. Tran, Michael S. Oh, Camelia Dumitras, William P. Crosson, Rui Li, Tejas S. Patel, Samantha Man, Cara E. Yean, Jensen Abascal, ZiLing Huang, Stephanie L. Ong, Kostyantyn Krysan, Steven M. Dubinett, Bin Liu

**Affiliations:** 1 Division of Pulmonary and Critical Care, Department of Medicine, David Geffen School of Medicine at University of California, Los Angeles, Los Angeles, CA 90095, USA; 2 Department of Molecular and Medical Pharmacology, David Geffen School of Medicine at University of California, Los Angeles, Los Angeles, CA 90095, USA; 3 Department of Medicine, VA Greater Los Angeles Healthcare System, Los Angeles, CA 90073, USA; 4 Division of Hematology and Oncology, Department of Medicine, David Geffen School of Medicine at University of California, Los Angeles, Los Angeles, CA 90095, USA; 5 Molecular Biology Institute, University of California, Los Angeles, Los Angeles, CA 90095, USA; 6 Department of Pathology and Laboratory Medicine, David Geffen School of Medicine at University of California, Los Angeles, Los Angeles, CA 90095, USA; 7 Jonsson Comprehensive Cancer Center, University of California, Los Angeles, Los Angeles, CA 90095, USA

**Keywords:** dendritic cells, CXCL9, CXCL10, T cells, checkpoint blockade, NSCLC, CXCR3, *in situ* vaccination, immunosuppression, systemic immunity

## Abstract

Immune checkpoint blockade (ICB) with PD-1/PD-L1 inhibition has revolutionized the treatment of non-small cell lung cancer (NSCLC). Durable responses, however, are observed only in a subpopulation of patients. Defective antigen presentation and an immunosuppressive tumor microenvironment (TME) can lead to deficient T cell recruitment and ICB resistance. We evaluate intratumoral (IT) vaccination with CXCL9- and CXCL10-engineered dendritic cells (CXCL9/10-DC) as a strategy to overcome resistance. IT CXCL9/10-DC leads to enhanced T cell infiltration and activation in the TME and tumor inhibition in murine NSCLC models. The antitumor efficacy of IT CXCL9/10-DC is dependent on CD4^+^ and CD8^+^ T cells, as well as CXCR3-dependent T cell trafficking from the lymph node. IT CXCL9/10-DC, in combination with ICB, overcomes resistance and establishes systemic tumor-specific immunity in murine models. These studies provide a mechanistic understanding of CXCL9/10-DC-mediated host immune activation and support clinical translation of IT CXCL9/10-DC to augment ICB efficacy in NSCLC.

## Highlights

• Intratumoral (IT) CXCL9/10-DC inhibits tumor growth in NSCLC murine models
• IT CXCL9/10-DC induces T cell infiltration and activation inside the tumor
• IT CXCL9/10-DC enhances the antitumor efficacy of immune checkpoint blockade
• IT CXCL9/10-DC and anti-PD-1 combination leads to tumor-specific immune memory

## Graphical abstract

**Figure:**
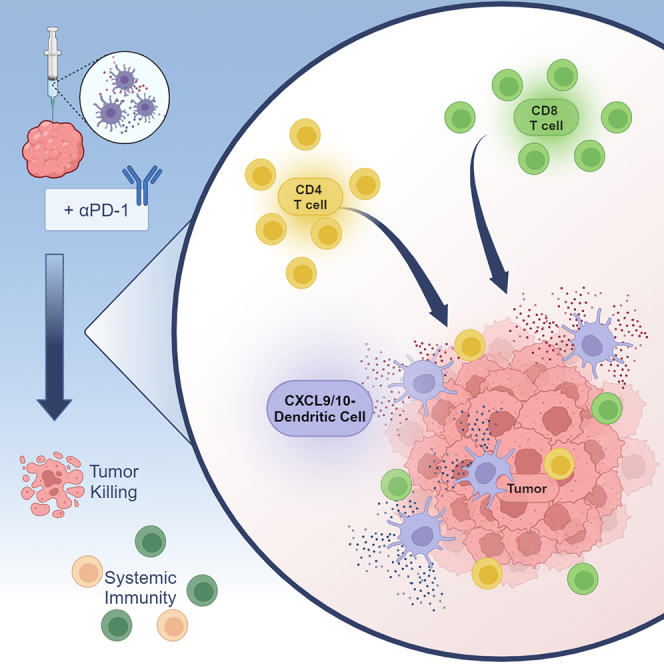

## Teaser

Lim et al. report intratumoral (IT) administration of chemokine genes CXCL9- and CXCL10-engineered dendritic cells (CXCL9/10-DC) as a potential therapy for non-small cell lung cancer (NSCLC). IT CXCL9/10-DC promotes T cell infiltration and activation in the tumor microenvironment and enhances antitumor efficacy of immune checkpoint blockade in murine NSCLC models.

## Introduction

Recent advances in immunotherapy, including immune checkpoint blockade (ICB) with programmed death-1/programmed death-ligand 1 (PD-1/PD-L1) inhibition, have revolutionized the treatment of non-small cell lung cancer (NSCLC), resulting in durable responses and improved overall survival in a subset of patients.^1^^,^^2^^,^^3^^,^^4^ However, the majority of patients do not respond to ICB monotherapy, and many have disease progression after an initial response^5^ in which setting options are limited.^6^^,^^7^^,^^8^^,^^9^ Successful clinical responses to PD-1/PD-L1 blockade are often associated with an increased baseline expression of PD-L1 and preexisting T cell infiltration in the tumor microenvironment (TME).^10^^,^^11^^,^^12^

Chemokines are essential soluble mediators that facilitate the recruitment of T cells into the TME.^13^ The C-X-C motif chemokine ligand 9 (CXCL9) and CXCL10, which are interferon γ (IFN-γ)-inducible chemokines, are predominantly secreted by tumor-residing CD103^+^ dendritic cells (DCs) and tumor-associated macrophages.^14^^,^^15^^,^^16^^,^^17^ CXCL9 and CXCL10 (CXCL9/10) signal through C-X-C motif chemokine receptor 3 (CXCR3) to promote the tumor infiltration of CXCR3^+^ effector T cells, including type 1 T helper (Th1) cells and CD8^+^ cytotoxic T lymphocytes.^18^ In addition to facilitating effector T cell infiltration into the tumor, the CXCL9/CXCL10/CXCR3 signaling cascade is required for optimal T cell activation.^14^^,^^16^^,^^17^^,^^19^ A recent landmark pan-cancer metadata analysis identified CXCL9 expression as one of the strongest predictors of response to ICB in cancer patients.^20^ This observation aligns with studies demonstrating that high expression of CXCL9, CXCL10, and/or CXCR3 in tumor biopsies is associated with improved overall survival in patients treated with ICB.^16^^,^^21^ Collectively, these findings underscore the essential role of CXCL9/10 in the TME as mediators of ICB-induced antitumor immunity.

DC *in situ* vaccination has emerged as a promising approach to overcome resistance to ICB.^22^^,^^23^ Intratumoral (IT) vaccination provides DCs access to the full repertoire of available tumor antigens to facilitate broad antitumor T cell responses. In preclinical studies, we and others have shown that genetically modified DC vaccines that secrete chemokines can condition the soluble and cellular mediators in the TME to facilitate favorable antitumor responses.^24^^,^^25^^,^^26^^,^^27^ Notably, cytokine gene-engineered DCs outperformed fibroblasts as an expression vehicle, underscoring the importance of the antigen presentation function of DCs for optimal efficacy.^24^ Given the pivotal role of CXCL9/10 in facilitating T cell-mediated antitumor immune responses, this study evaluates the efficacy of *in situ* vaccination with CXCL9/10-engineered DC (CXCL9/10-DC) to enhance the efficacy of ICB immunotherapies in murine NSCLC models.

We find that IT CXCL9/10-DC augments T cell infiltration and activation in the TME, leading to effective tumor inhibition in multiple syngeneic murine NSCLC models. The antitumor efficacy of IT CXCL9/10-DC is dependent on both CD4^+^ and CD8^+^ T cells, as well as CXCR3-mediated T cell trafficking and T cell egress from the lymph nodes (LNs). IT CXCL9/10-DC overcomes resistance to PD-1/PD-L1 blockade in a *Lkb1*-deficient *Kras*-mutant murine NSCLC model with low tumor mutational burden (TMB).^28^ In addition, IT CXCL9/10-DC enhances the relatively modest efficacy of PD-1/PD-L1 blockade in a *Lkb1*-deficient, *Kras*-mutant murine NSCLC model with high TMB, leading to the complete eradication of a subset of tumors and the establishment of tumor-specific immune memory. These findings provide evidence for the potential clinical translation of IT CXCL9/10-DC as a strategy to overcome resistance and enhance clinical efficacy of ICB immunotherapy in NSCLC.

## Results

### The CXCL9/10 and CXCR3 axis correlates with tumor infiltration of immune-activating cell subtypes in human NSCLC

**Figure 1:**
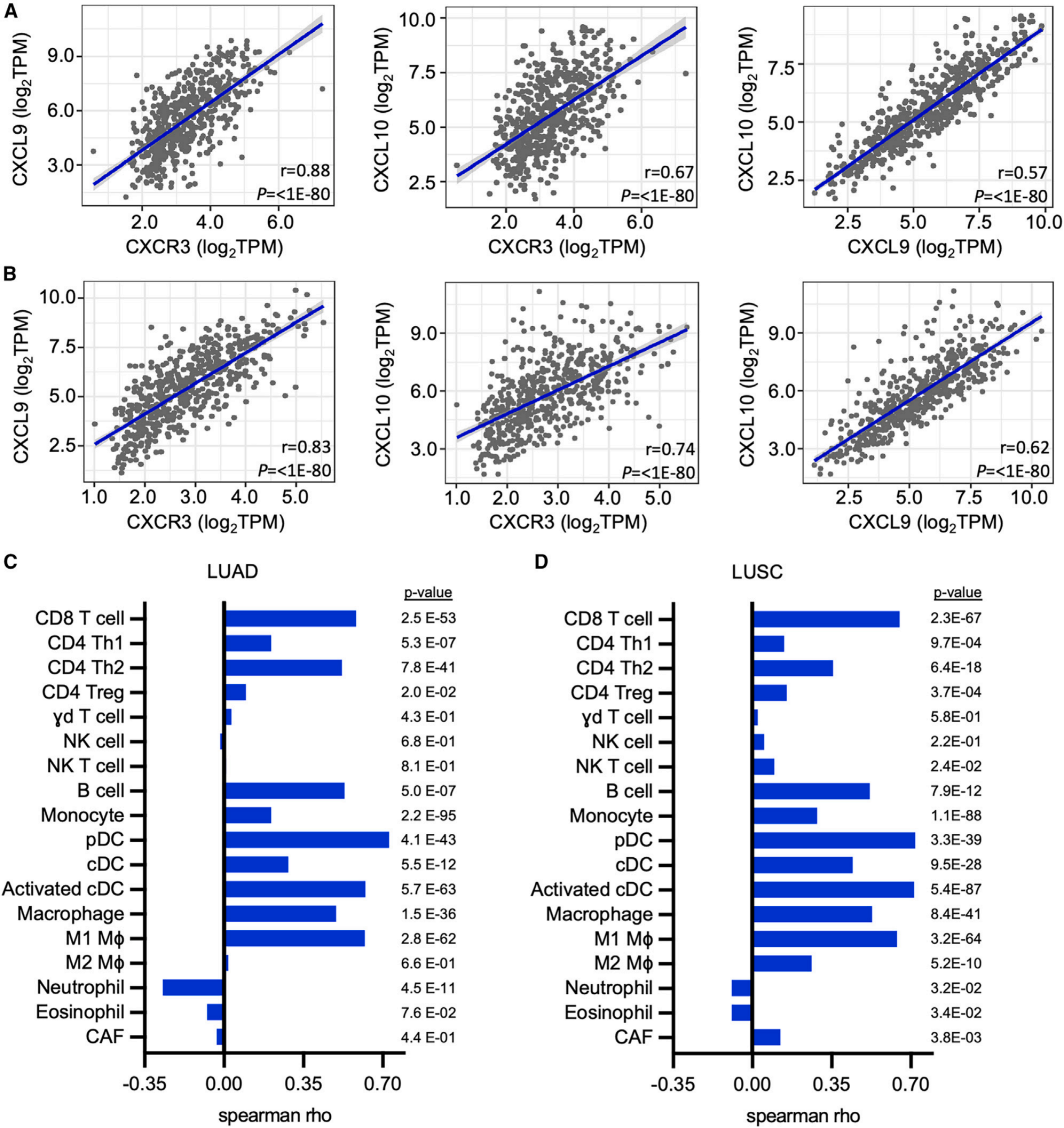
The Spearman correlations between the expression levels of *CXCL9*, *CXCL10*, and *CXCR3* and immune cell infiltration in human NSCLC derived from the TCGA database using xCell expression signatures (A and B) Correlations between *CXCL9*, *CXCL10*, and *CXCR3* expression levels in both (A) LUAD and (B) LUSC. (C) Correlation table between CXCL9/10 and various immune cell signatures in LUAD. (D) Same as in (C), except for LUSC.

Analysis of NSCLC data from The Cancer Genome Atlas (TCGA) identified a strong correlation (Spearman correlation coefficient >0.55) among the expression of *CXCL9, CXCL10*, and their cognate receptor (*CXCR3*) in both lung adenocarcinoma (LUAD) and lung squamous carcinoma (LUSC) (Figures 1A and 1B). Immune infiltration profiles derived from gene expression by xCELL^29^ and TIMER^30^ approaches were used to determine the associations between immune infiltration profiles and the average expression of *CXCL9/10* (Figures 1C and 1D). Among lymphocytes, tumor *CXCL9/10* expression in both LUAD and LUSC showed strong positive correlations with the infiltration of both CD8^+^ T cells and CD4^+^ Th2 effector T cells and a weak association with CD4^+^ Th1 effector T cells, consistent with the established role of the CXCL9/10-CXCR3 axis in T cell recruitment^14^^,^^16^ (Figures 1C, 1D, and S1A). A strong association with B cell infiltration, some known to express CXCR3, was also observed, consistent with immune activation.^31^ Within the myeloid compartment, CXCL9/10 expression positively correlated with both plasmacytoid DC (pDC) and monocytes, which are known to express CXCR3. A correlation was also found with conventional DC (cDC) infiltration in both LUAD and LUSC, with the strongest association observed with activated DCs (Figures 1C, 1D, and S1B). CXCL9/10 expression showed a positive correlation with infiltrating macrophages, specifically the M1 subtype, but not the M2 subtype (Figures 1C, 1D, and S1B). These data are consistent with previous reports demonstrating that cDCs and macrophages are the predominant cell types that secrete CXCL9/10.^14^^,^^16^ Other cell subtypes, including cancer-associated fibroblasts, natural killer (NK), NKT cells, neutrophils (Neu), and eosinophils, showed no or weak correlations with *CXCL9/10* expression in LUAD and LUSC. These findings support the notion that CXCL9/10 expression in the TME is associated with an antitumor immune signature.

### *In situ* vaccination with CXCL9/10-DC inhibits lung cancer in preclinical murine models

To generate CXCL9/10-DC, bone marrow-derived DCs (BMDCs)^24^^,^^27^ were transduced with a lentiviral construct encoding CXCL9 or CXCL10 (Figure S2A; STAR Methods). Transduced CXCL9-DC and CXCL10-DC secreted 15–22 ng/mL of the corresponding chemokine per million cells after 24 h *in vitro* culture (Figure S2B). Equal numbers of transduced CXCL9-DC and CXCL10-DC were combined to generate CXCL9/10-DC. CXCL9/10 transduction did not alter the phenotype of BMDC (CD11c^+^CD11b^+^MHCII^hi^), whereas a minor increase of the activation marker CD86 was observed (Figure S2C). CXCL9/10 DCs retained phagocytic ability, as determined by the fluorescein isothiocyanate-dextran uptake assay (Figure S2D).

**Figure 2:**
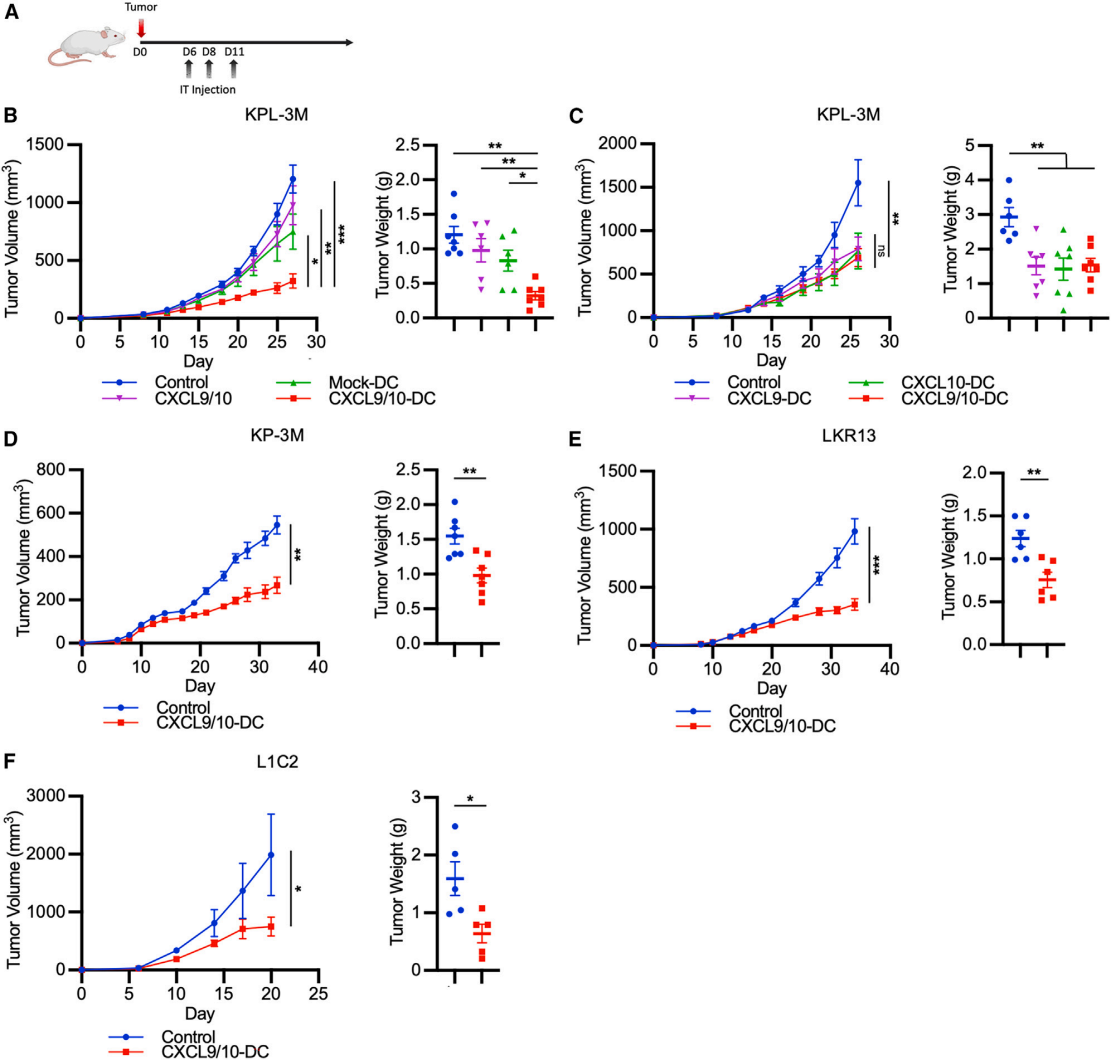
*In situ* vaccination with CXCL9/10-DC inhibits murine lung cancer (A) A schematic of *in vivo* mouse efficacy studies. On D6 post-s.c. tumor inoculation, mice bearing ∼50-mm^3^ tumors were randomized and subjected to treatments as detailed in STAR Methods via IT injections on D6, D8, and D11. Tumor volumes were recorded every 2–3 days and tumor weights were measured on the day of euthanasia. (B) FVB mice were inoculated with *Kras*^*G12D*^*Tp53*^*−/−*^*Lkb1*^*−/−*^ (KPL)-3M tumor cells (1.25 × 10^5^ cells) and treated with PBS control; CXCL9 and CXCL10 recombinant proteins (CXCL9/10) at 20 ng each per injection; vector-transduced DC (Mock-DC, 2 × 10^6^ per injection); or CXCL9/10-DC (1 × 10^6^ CXCL9-DC and CXCL10-DC each per injection) (n = 6–8 mice per group). (C) FVB mice were inoculated with KPL-3M tumor cells and treated with PBS control; CXCL9-DC (2 × 10^6^/injection); CXCL10-DC (2 × 10^6^/injection); or CXCL9/10-DC as in (B). (D) FVB mice were inoculated with *Kras*^*G12D*^*Tp53*^*−/−*^ (KP)-3M tumor cells (2 × 10^6^ cells) and treated with PBS control or CXCL9/10-DC as in (B). (E)129-E mice were inoculated with LKR13 (*Kras*^G12D^) tumor cells (2 × 10^6^ cells) and treated as in (D). (F) BALB/c mice were inoculated with L1C2 tumor cells (1 × 10^6^ cells) and treated as in (D) (n = 5–6 mice per group). Error bars represent SEM. p values were determined by one-way ANOVA adjusting for multiple comparisons for (B) and (C), and two-tailed t test for (D–F). ∗p < 0.05; ∗∗p < 0.005; ∗∗∗p < 0.0005.

The antitumor efficacy of *in situ* vaccination with CXCL9/10-DC was assessed in multiple syngeneic murine models of lung cancer (Figure 2A). *LKB1*-inactivating mutations in *KRAS*-mutant NSCLC are associated with a suppressed state of cell-mediated immunity and drive resistance to ICB.^32^^,^^33^^,^^34^ IT CXCL9/10-DC vaccination significantly inhibited the growth of *Kras*^*G12D*^*Tp53*^*−/−*^*Lkb1*^*−/−*^-3M (KPL-3M) tumors harboring high TMB (7.2 mutations/Mb).^28^ IT PBS control, recombinant CXCL9/10 chemokines, and vector control virus-transduced DCs (Mock-DC) did not provide antitumor efficacy (Figure 2B). IT CXCL9-DC or CXCL10-DC provided similar efficacy as compared to CXCL9/10-DC in the KPL-3M tumor-bearing mice (Figure 2C). We had previously shown that the depletion of either CXCL9 or CXCL10 inhibits antitumor responses in murine lung cancer, underscoring nonredundant functions of these cytokines in cancer immunity.^35^ In addition, recent studies have highlighted the importance of both CXCL9^16^ and CXCL10^14^ in mediating antitumor immunity independently. Therefore, we proceeded with a combined CXCL9/10-DC vaccination approach. CXCL9/10-DC vaccination mitigated tumor growth in three additional syngeneic murine lung cancer models, including high TMB *Kras*^G12D^*Tp53*^*−/−*^-3M (KP-3M; 22.1 mutations/Mb), low TMB *Kras*^G12D^ (LKR13; 0.7 mutations/Mb),^28^ and bronchoalveolar carcinoma (L1C2) (Figures 2D–2F).

### CXCL9/10-DC-induced T cell recruitment and activation are requisite for antitumor efficacy

*In vivo* trafficking of CXCL9/10-DC following a single IT injection was investigated by flow cytometry following CellTracker Red labeling. Mock-DC was included as a control. Following IT vaccination, a continuous decrease in the frequency of CXCL9/10-DC and Mock-DC was observed over time, which was predominantly a result of decreased viability (Figure S3A). Tumor vaccination with CXCL9/10-DC induced a statistically significant elevation in the concentrations of CXCL9 and CXCL10 within the TME at 24 h, surpassing levels observed with PBS control and Mock-DC (Figure S3B). This increase was transient, because the levels of CXCL9 and CXCL10 declined at 48 h, aligning with the temporal decline of the injected DCs in the TME (Figures S3A and S3B). The kinetics of *in vivo* DC viability and CXCL9/10 expression in the TME support our treatment regimen of multiple IT injections every 2–3 days (Figure 2A). Although the DC vaccines had a limited lifespan, IT CXCL9/10-DC induced infiltration of both endogenous cDC1s and cDC2s into the tumor at 24 h (Figure S3C). This was followed by an increase in both CD4^+^ and CD8^+^ T cells in the TME at 48 h (Figure S3D). No differences in the expression of CXCR3 were observed within the T cell compartment of the TME at this early time point (Figure S3E). These results indicate that CXCL9/10-DC vaccination promotes infiltration of endogenous DCs and T cells into the tumor.

**Figure 3:**
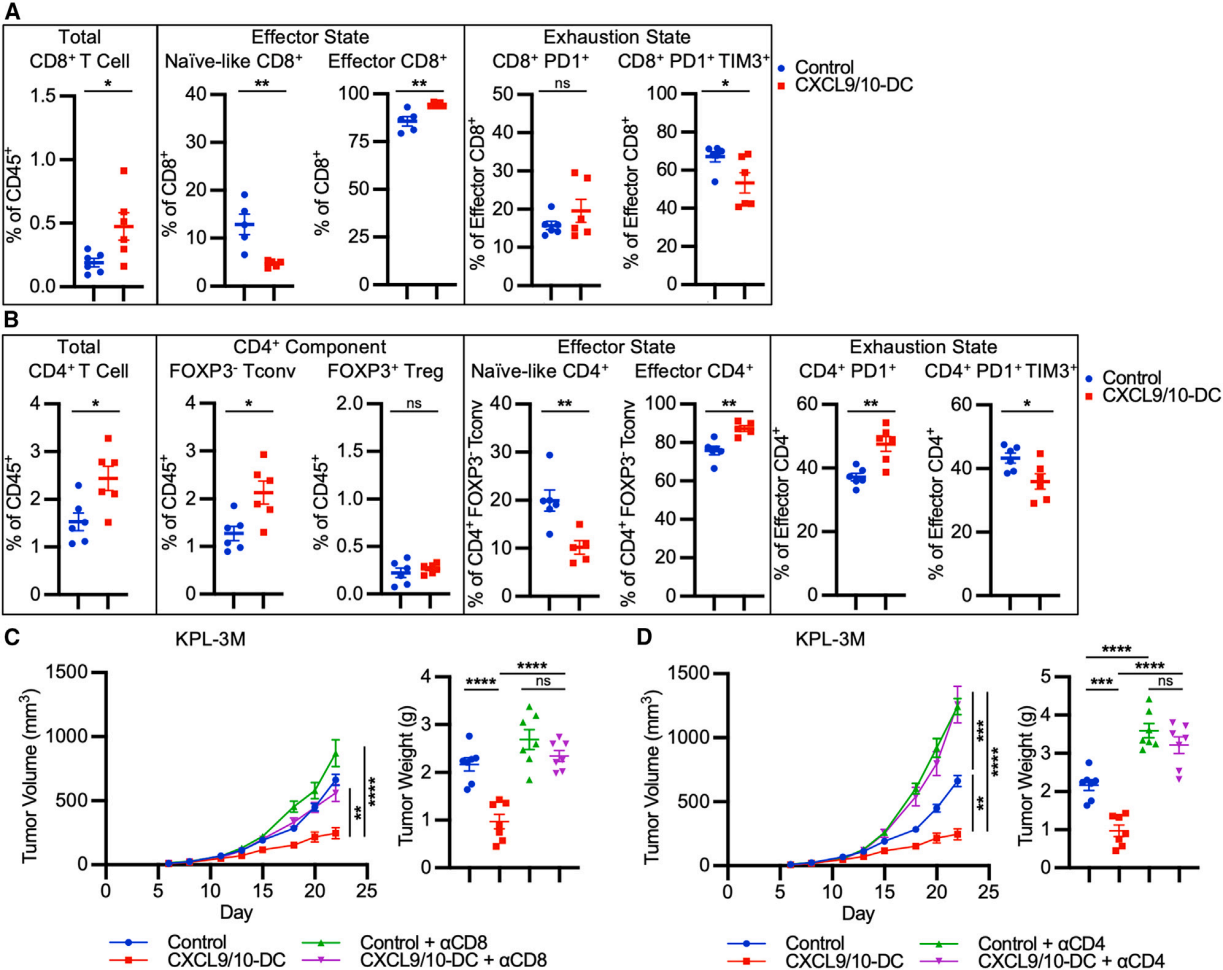
*In situ* vaccination with CXCL9/10-DC facilitates T cell recruitment and activation required for the antitumor efficacy (A) CXCL9/10-DC therapy-induced changes in CD8^+^ T cells. Tumors were collected on D14 post-inoculation (1.25 × 10^5^ KPL-3M inoculated s.c. in FVB mice) following treatment with PBS control or CXCL9/10-DC (IT 1 × 10^6^ CXCL9-DC and CXCL10-DC each per injection on D6, D8, and D11) (n = 5–6 mice per group). CD8 naive-like, CD8^+^CD44^−^CD62L^+^; CD8 effector, CD8^+^CD44^+^CD62L^−^. (B) CXCL9/10-DC therapy-induced changes in CD4^+^ T cells revealed by flow cytometry as in (A). CD4 Tconv, CD4^+^FOXP3^−^; CD4 Treg, CD4^+^FOXP3^+^; CD4 naive-like, CD4^+^CD44^−^CD62L^+^; CD4 effector, CD4^+^CD44^+^CD62L^−^. (C) CD8^+^ T cells are required for CXCL9/10-DC-driven antitumor efficacy. On D6 post-tumor inoculation (1.25 × 10^5^ KPL-3M inoculated s.c.), FVB mice bearing ∼50-mm^3^ tumors were randomized and treated with PBS + isotype control; CXCL9/10-DC (IT 1 × 10^6^ CXCL9-DC and CXCL10-DC each per injection on D6, D8, and D11) + isotype; PBS + anti-mouse CD8 (200 μg i.p. injection every 2 days starting on D6 until the end of the study); or CXCL9/10-DC + anti-mouse CD8 as detailed above (n = 6–8 mice per group). (D) CD4^+^ T cells are required for CXCL9/10-DC-driven antitumor efficacy. Same as in (C), except that anti-mouse CD4 was used. Error bars represent SEM. p values were determined by two-tailed t test for (A) and (B) and one-way ANOVA adjusting for multiple comparisons for (C) and (D). n.s., not significant; ∗p < 0.05; ∗∗p < 0.005, ∗∗∗p < 0.0005, ∗∗∗∗p < 0.00005.

To evaluate the downstream effects of CXCL9/10-DC on mediators of antitumor immunity in the TME, flow phenotyping was performed on day 14 (D14) following IT CXCL9/10-DC or PBS control on D6, D8, and D11 (Figures S4A and S4B). IT CXCL9/10-DC promoted an increase in CD8^+^ T cell infiltration into the tumor compared to the control (Figure 3A). Within the CD8^+^ T cells, CXCL9/10-DC vaccination led to an increase in the effector cells (CD62L^−^CD44^+^) and a decrease in terminally exhausted PD-1^+^TIM3^+^ CD8^+^ effector T cells (Figure 3A). A significant increase in total CD4^+^ T cells was also observed in the TME following CXCL9/10-DC treatment, driven by an increase in CD4^+^FOXP3^−^ T conventional helper (Tconv) cells, with no changes observed in regulatory T cells (Treg; CD4^+^FOXP3^+^) (Figure 3B). Within the Tconv cells, CXCL9/10-DC treatment induced a shift from naive-like (CD62L^+^CD44^−^) to an effector (CD62L^−^CD44^+^) state, similar to that in CD8^+^ T cells (Figure 3B). CXCL9/10-DC vaccination induced an increase in PD-1^+^ activated CD4^+^FOXP3^−^ effector T cells with a concurrent decrease in PD-1^+^TIM3^+^ terminally exhausted T cells (Figure 3B).^36^^,^^37^ A statistically significant increase in NKT but not NK cells was observed following CXCL9/10-DC therapy (Figure S4C). No significant changes were noted in monocytes, macrophages, cDC1s, cDC2s, or Neu (Figure S4D). These data suggest that following an induction of endogenous cDC into the tumor, the downstream mediators of IT CXCL9/10-DC are predominantly T cells.

To evaluate the functional importance of endogenous T cells following CXCL9/10-DC vaccination, CD8 and CD4 depletion studies were performed with blocking antibodies. CD8^+^ T cell depletion abolished the antitumor efficacy of IT CXCL9/10-DC (Figures 3C and S4E). CD4^+^ T cell depletion led to increased tumor growth in the control group, supporting the essential role of CD4^+^ T cells in controlling tumor progression (Figures 3D and S4F). CD4^+^ T cell depletion completely abrogated the antitumor efficacy of CXCL9/10-DC. These data demonstrate that the antitumor efficacy of CXCL9/10-DC vaccination is dependent on both endogenous CD8^+^ and CD4^+^ T cells.

### T cell recruitment from LNs is required for CXCL9/10-DC-mediated antitumor efficacy

**Figure 4:**
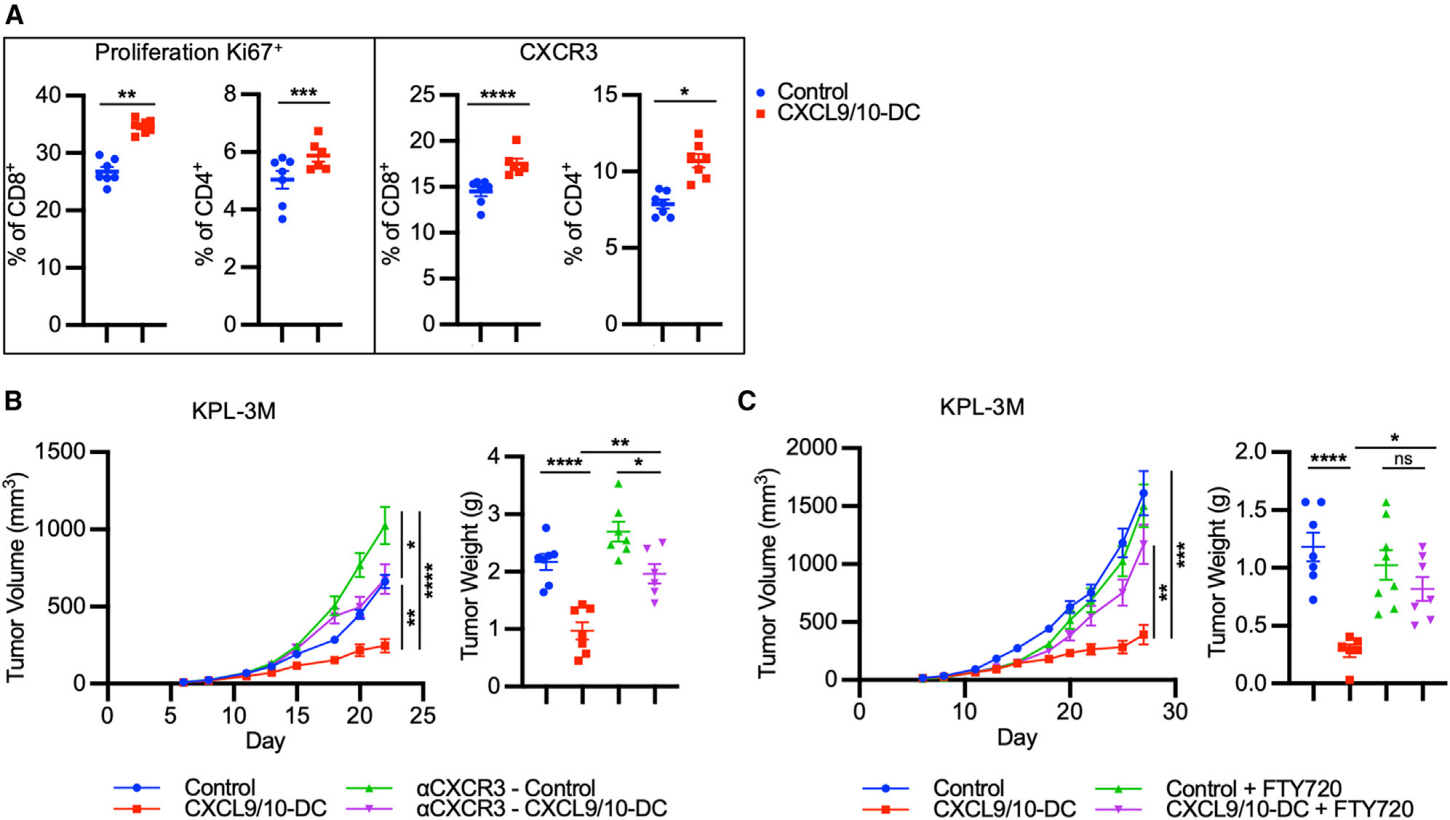
T cell recruitment from LNs is essential for CXCL9/10-DC-mediated antitumor efficacy (A) Flow phenotyping of T cells in TDLNs following CXCL9/10-DC therapy. TDLNs were collected on D16 post-inoculation (1.25 × 10^5^ KPL-3M delivered s.c. in FVB mice) following treatment with PBS control or CXCL9/10-DC (IT 1 × 10^6^ CXCL9-DC and CXCL10-DC each per injection on D6, D8, and D11) (n = 6–8 mice per group). (B) CXCR3^+^ T cells are required for CXCL9/10-DC-mediated antitumor efficacy. On D6 post-tumor inoculation (1.25 × 10^5^ KPL-3M delivered s.c. in FVB mice), mice bearing ∼50-mm^3^ tumors were randomized and treated with PBS + isotype; CXCL9/10-DC (IT 1 × 10^6^ CXCL9-DC and CXCL10-DC each per injection on D6, D8, and D11) + isotype; PBS + anti-CXCR3 (i.p. at 200 μg/injection) every other day until the end of the study; or CXCL9/10-DC + anti-CXCR3 as above (n = 6–8 mice per group). (C) T cell migration from LNs is required for CXCL9/10-DC-mediated antitumor efficacy. Same as in (B), except that fingolimod (FTY720) at 2 mg/kg (i.p. every other day) instead of anti-CXCR3 was used. Error bars represent SEM. p values were determined by two-tailed t test for (A) and one-way ANOVA adjusting for multiple comparisons for (B) and (C). ∗p < 0.05; ∗∗p < 0.005; ∗∗∗p < 0.0005; ∗∗∗∗p < 0.00005.

Recent reports have demonstrated that *in situ* vaccination with BMDCs facilitates the activation of *de novo* T cell responses by endogenous cDC1s in tumor draining LNs (TDLNs).^38^ Evaluation of TDLNs following IT CXCL9/10-DC therapy in KPL-3M tumor-bearing mice showed enhanced proliferation of both CD4^+^ and CD8^+^ T cells, ascertained by Ki67^+^ staining, and an increased T cell effector polarization, determined by CXCR3 expression (Figure 4A). To assess the dependency of IT CXCL9/10-DC efficacy on the CXCL9/CXCL10/CXCR3 axis, which mediates effector T cell recruitment, efficacy studies were performed in the presence of CXCR3-neutralizing antibody.^39^ Treatment of mice bearing KPL-3M tumors intraperitoneally (i.p.) with CXCR3 blockade enhanced tumor growth compared to the isotype control (Figure 4B). CXCR3 blockade resulted in a partial reduction of the antitumor efficacy of IT CXCL9/10-DC, compared to the IT vehicle control. We then used fingolimod (FTY720), a sphingosine 1-phosphate inhibitor that blocks T cell egress from the LNs^17^ and observed that FTY720 abolished the efficacy of IT CXCL9/10-DC (Figure 4C). These data demonstrate that IT CXCL9/10-DC-mediated antitumor efficacy is dependent on the CXCR3 axis as well as T cell egress from the LNs into the tumor.

### Spatial analysis of T cell recruitment following CXCL9/10-DC treatment

**Figure 5:**
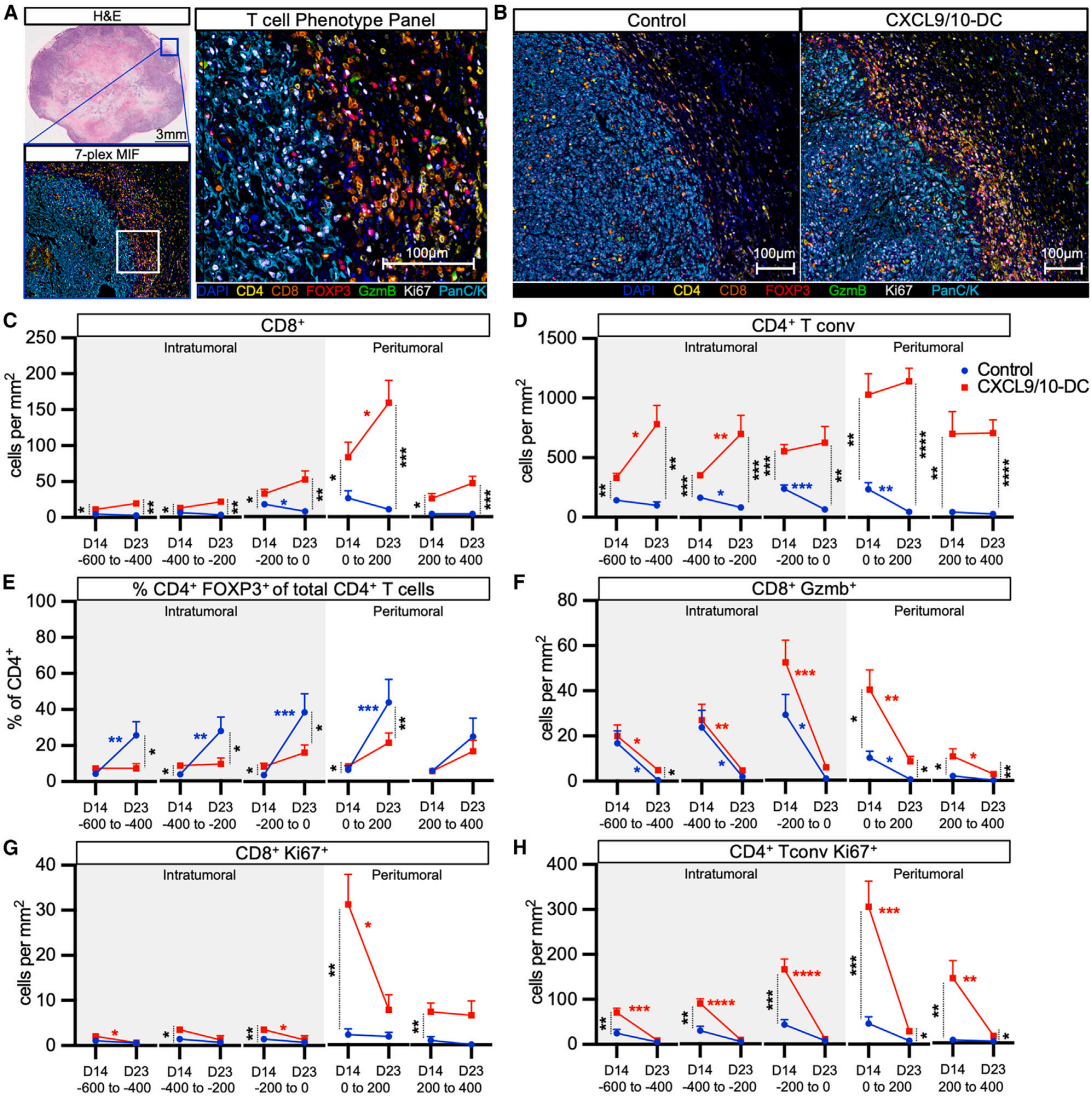
Spatial analysis of T cell recruitment following CXCL9/10-DC treatment by MIF Tumors were collected on D14 and D23 post-inoculation (1.25 × 10^5^ KPL-3M delivered s.c. in FVB mice) following treatment with PBS control or CXCL9/10-DC (IT 1 × 10^6^ CXCL9-DC and CXCL10-DC each per injection on D6, D8, and D11) (n = 6–8 mice per group). Tumor tissues were fixed and embedded for MIF staining, followed by quantification using inForm software (Akoya). (A) A representative image of a tumor section stained with a 7-plex MIF panel (CD4, CD8, GzmB, FOXP3, Ki67, PanC/K, and DAPI). (B) Representative images of D23 control and CXCL9/10-DC-treated tumors stained with the same panel as in (A). (C–H) Changes in the enumeration of CD8^+^ T cells (C), CD4^+^ T cells (D), %CD4^+^FOXP3^+^ T cells of total CD4^+^ T cells (E), GzmB^+^ CD8^+^ T cells (F), Ki67^+^ CD8^+^ (G), and Ki67^+^ CD4^+^ T cells (H), following treatment with control (blue) or CXCL9/10-DC (red) from D14 and D23 at 200-μm intervals relative to the tumor border (n = 6–8 mice per group). The intratumoral space is shaded in contrast to the peritumoral space. Error bars represent SEM. p values were determined by two-tailed t test for (C–H). ∗p < 0.05; ∗∗p < 0.005; ∗∗∗p < 0.0005; ∗∗∗∗p < 0.00005. Colored asterisks over the bar represent the statistics for longitudinal changes from D14 to D23 of each group.

To evaluate the spatial distribution of endogenous T cells within the TME, multiplex immunofluorescence (MIF) was performed on D14 and D23 tumors using a 7-plex panel (CD4, CD8, FOXP3, granzyme B [GzmB; activation], Ki67 [proliferation], PanC/K [tumor marker], and DAPI [DNA marker]) (Figure 5A). Analysis of stained tumor sections revealed the greatest accumulation of immune cells at the edge of the tumor following IT CXCL9/10-DC (Figure 5B). Whole-slide quantification showed a significant increase in CD8^+^ T cells in both the intratumoral and peritumoral regions following CXCL9/10-DC therapy compared to PBS control (Figures 5C and S5). Although a progressive decrement in CD8^+^ T cell tumor infiltration was observed in control mice from D14 to D23, CXCL9/10 vaccination induced progressive recruitment of CD8^+^ T cells to the tumor margin over time (Figure 5C). In accordance with results obtained by flow cytometry (Figure 3), MIF showed a 4- to 5-fold increase of CD4^+^ T cells in the TME as compared to CD8^+^ T cells (Figure 5D). IT CXCL9/10-DC led to enhanced and progressive accumulation of CD4^+^ Tconv cells (CD4^+^FOXP3^−^) from D14 to D23 compared to vehicle control (Figure 5D). In contrast, control mice had a cumulative decline in tumor-infiltrating CD4^+^ Tconv cells and a concurrent increase in Tregs on D23 compared to D14 (Figures 5D and 5E). IT CXCL9/10-DC led to an increased depth of tumor penetration by CD4^+^ T cells when compared to CD8^+^ T cells (Figure 5D). In contrast to total CD8^+^ T cells, which had the highest accumulation in the 0- to 200-μm peritumoral region, the largest density of CD8^+^ GzmB^+^ T cells was observed in the 0- to 200-μm intratumoral region on D14 (Figure 5F), suggesting antitumor cytolytic activity at the tumor border.^19^^,^^40^^,^^41^ Previous studies have shown the importance of the localization of CD8^+^ T cells at the tumor border in predicting responses to ICB.^11^ We observed a marked reduction in both GzmB^+^ and Ki67^+^ CD8^+^ T cells as well as Ki67^+^ CD4^+^ Tconv cells on D23 compared to D14 following IT CXCL9/10-DC (Figures 5F–5H), consistent with a progressive decline in T cell proliferation and activity, likely due to enhanced immunosuppression in the TME.

### CXCL9/10-DC potentiates ICB immunotherapy in LKB1-deficient NSCLC murine models with varying TMB

**Figure 6:**
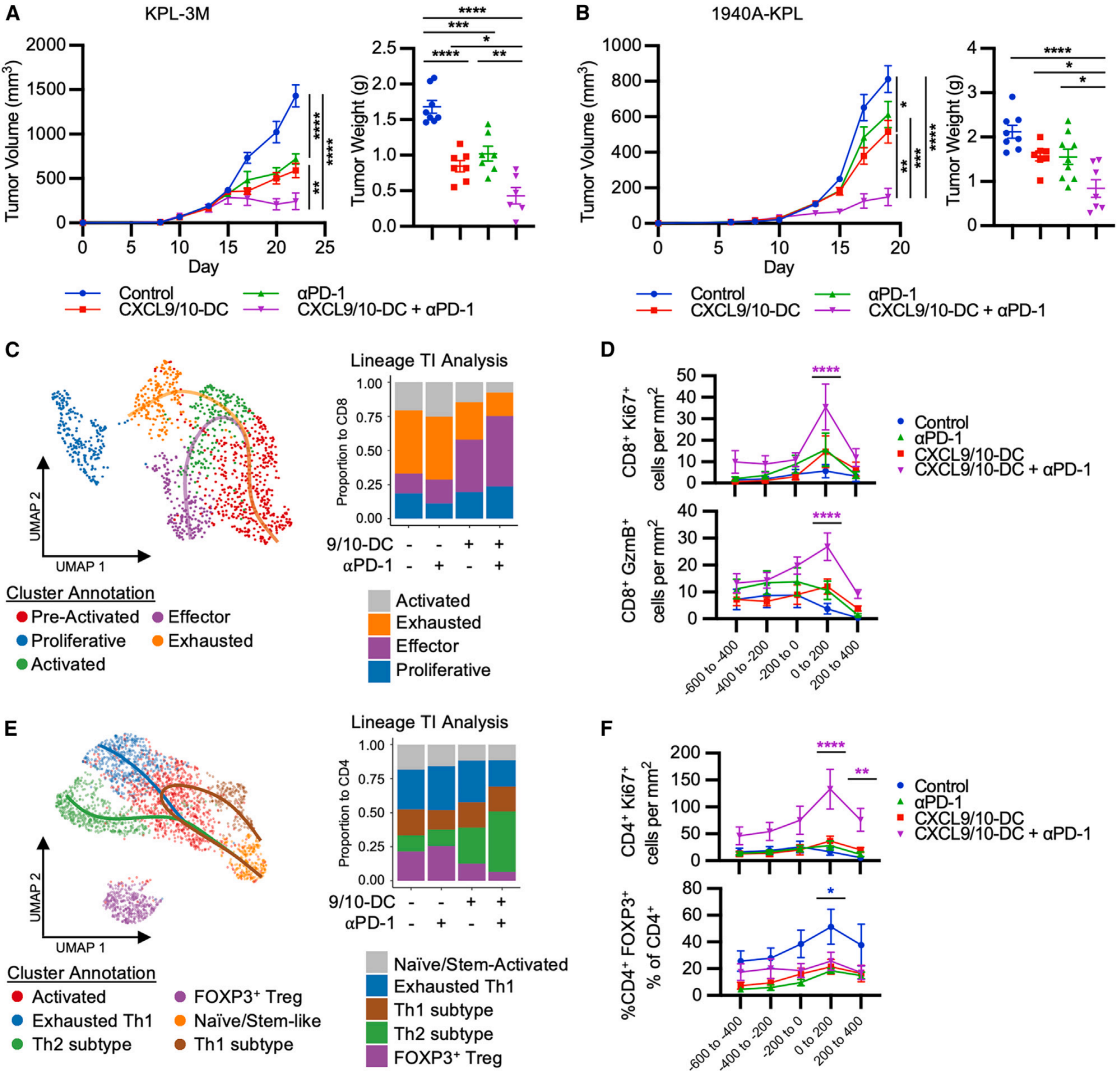
CXCL9/10-DC potentiates ICB immunotherapy in murine NSCLC models (A) CXCL9/10-DC enhances the efficacy of anti-PD-1. On D6 post-tumor inoculation (1.25 × 10^5^ KPL-3M delivered s.c.), FVB mice bearing ∼50-mm^3^ tumors were randomized and treated with PBS control; CXCL9/10-DC (IT 1 × 10^6^ CXCL9-DC and CXCL10-DC each per injection on D6, D8, and D11); anti-PD-1 (i.p. injections at 200 μg/injection on D6, D8, D11, and D14); or the combination of CXCL9/10-DC and anti-PD-1 (n = 6–8 mice per group). Tumor growth curves and tumor weights at the time of euthanasia are presented. (B) Same as in (A), except that mice were s.c. inoculated with 1 × 10^5^ 1940A-KPL tumor cells. (C) In-depth scRNA-seq analysis identifies distinct CD8^+^ T cell subtypes (cells pooled from 5 tumors per group). Lineage trajectory interference (TI) analysis of CD8^+^ T cell changes following various treatments. (D) Spatial quantification of CD8^+^Ki67^+^ and CD8^+^GzmB^+^ T cells were performed on D23 per Figure S6 (n = 6–8 mice per group). (E) Therapy-induced changes in CD4^+^ T cells revealed by scRNA-seq. Same as in (C). (F) Spatial quantification of CD4^+^Ki67^+^ and FOXP3^+^ Treg cells. Same as in (D). Error bars represent SEM. p values were determined by one-way ANOVA, adjusting for multiple comparisons for (A) and (B) and two-tailed t test for (D) and (F). ∗p < 0.05; ∗∗p < 0.005; ∗∗∗p < 0.0005; ∗∗∗∗p < 0.00005.

We hypothesized that the progressive decrease in T cell proliferation and cytolytic activity following IT CXCL9/10-DC monotherapy could be due to adaptive immune resistance mediated by the upregulation of checkpoint inhibitors within the TME. Combination therapy of IT CXCL9/10-DC and i.p. PD-1/PD-L1 inhibition was evaluated in two murine models of LKB1-deficient NSCLC with varying TMB.^28^ Treatment of KPL-3M (TMB high) tumor-bearing mice with i.p. anti-PD-1 or IT CXCL9/10-DC monotherapy provided modest antitumor efficacy, with no observed tumor eradication. The combination of CXCL9/10-DC and anti-PD-1 or anti-PD-L1 elicited robust tumor regression, resulting in complete eradication of tumors in ∼30% of the mice (Figures 6A and S6A). Histological evaluation of paraffin-embedded tumors demonstrated decreased tumor content following IT CXCL9/10-DC as monotherapy, or in combination with anti-PD-1, as determined by the ratio of PanC/K^+^ (tumor) areas to total tissue area (Figures S6B and S6C). In contrast to KPL-3M, which has a high TMB, anti-PD-1 therapy in mice bearing 1940A-KPL tumors with low TMB (1.7 mutations/Mb) did not alter tumor growth rates, consistent with prior reports.^28^ However, combination therapy with IT CXCL9/10-DC vaccination and anti-PD-1 resulted in robust antitumor responses, which were significantly greater than IT CXCL9/10 monotherapy (Figure 6B). Combination therapy also reduced metastatic tumor cell growth from lung digests compared to vehicle control, as determined by luciferase assay using the genetically engineered *Luc* in the 1940A-KPL tumor cells as a readout for cell growth, suggesting decreased pulmonary metastases in response to combination therapy (Figure S6D).

### Anti-PD-1 therapy augments CXCL9/10-DC vaccination-induced effector T cell proliferation and activity

Single-cell RNA sequencing (scRNA-seq) of sorted CD45^+^ tumor-infiltrating immune cells was conducted on D14 following IT CXCL9/10-DC and i.p. anti-PD-1 as monotherapies or in combination. Seven immune clusters were identified, including pDC, NK, T, B, Neu, and non-Neu myeloid (MoMacDC) cells (Figure S6E). An increase in T cells was accompanied by a decrease in the predominant Neu population following IT CXCL9/10-DC monotherapy or in combination with anti-PD-1, as compared to PBS control or anti-PD-1 alone (Figure S6E). Monotherapy with anti-PD-1 induced minimal changes in immune cell subtypes compared to control.

Within the CD8^+^ T cells, five sublineages—preactivated, activated, effector, exhausted, and proliferative—were identified (Figures 6C and S6F). Trajectory inference analysis, including cells from all of the clusters except those in the proliferative cluster, revealed that preactivated CD8^+^ T cells could evolve through two independent paths to either an effector or exhausted state (Figure 6C). IT CXCL9/10-DC as monotherapy or in combination with anti-PD-1 induced an enrichment of a CD8^+^ effector phenotype accompanied by a decrement of the exhausted state compared to control or anti-PD-1, with the highest magnitude of changes observed with combination therapy (Figure 6C). Flow cytometry confirmed increased IFN-γ and tumor necrosis factor α production in tumor-infiltrating CD8^+^ T cells in response to combination therapy compared to control, suggesting enhanced effector function (Figure S6G). The distribution of CD8^+^ T cells based on their differentiation paths was nearly identical between anti-PD-1 and the control groups (Figure 6C). We then used MIF to assess whether the addition of anti-PD-1 to IT CXCL9/10-DC monotherapy can augment the proliferation and cytolytic function of T cells, which was previously shown to diminish on D23 following IT CXCL9/10-DC monotherapy (Figures 5F–5H). The combination of anti-PD-1 with IT CXCL9/10-DC induced an overall increase in cytotoxic GzmB^+^CD8^+^ and proliferative Ki67^+^CD8^+^ T cells across all of the regions of the TME compared to monotherapies, with the most pronounced increase observed at the tumor border (0–200) (Figure 6D).

CD4^+^ T cells comprised six clusters, corresponding to naive/stem-like, activated, exhausted Th1, effector Tconv Th1 subtype, Th2 subtype, and Treg phenotypes (Figures 6E and S6H). Trajectory inference analysis revealed potential evolution of naive/stem-like CD4^+^ T cells through an activated state and subsequently three distinct paths resulting in exhausted Th1, Th1 subtypes, and Th2 subtypes. CXCL9/10-DC vaccination, as monotherapy or in combination, increased effector CD4^+^ T cells, which were predominantly driven by the Tconv Th2 phenotype, accompanied by a decrement of the Treg component compared to control or anti-PD-1 (Figure 6E). Combination therapy of CXCL9/10-DC and anti-PD-1 resulted in a decrease in exhausted Th1 when compared to the other groups (Figure 6E). Although anti-PD-1 therapy increased CD4^+^ T cell tumor infiltration, the sublineage composition was similar to control. Flow cytometry analysis confirmed that combination therapy augmented the production of both Th1 and Th2 cytokines compared to control (Figure S6I). MIF analysis demonstrated an overall increase in proliferative CD4^+^ T cells following combination therapy, whereas the vehicle control group showed an increase in Treg abundance across all regions of the tumor, with the most pronounced increase at the tumor border (0–200) (Figure 6F). These spatial data combined with studies from Figures 5E–5H, demonstrate that IT CXCL9/10-DC vaccination enhances T cell recruitment to the tumor margin, and the addition of anti-PD-1 promotes sustained proliferation and effector functions of tumor-infiltrating CD4^+^ and CD8^+^ T cells, which likely mediate the antitumor efficacy of combination therapy (Figures 6A and 6B).

### Combination treatment with CXCL9/10-DC and anti-PD-1 generates systemic tumor-specific immunity

**Figure 7:**
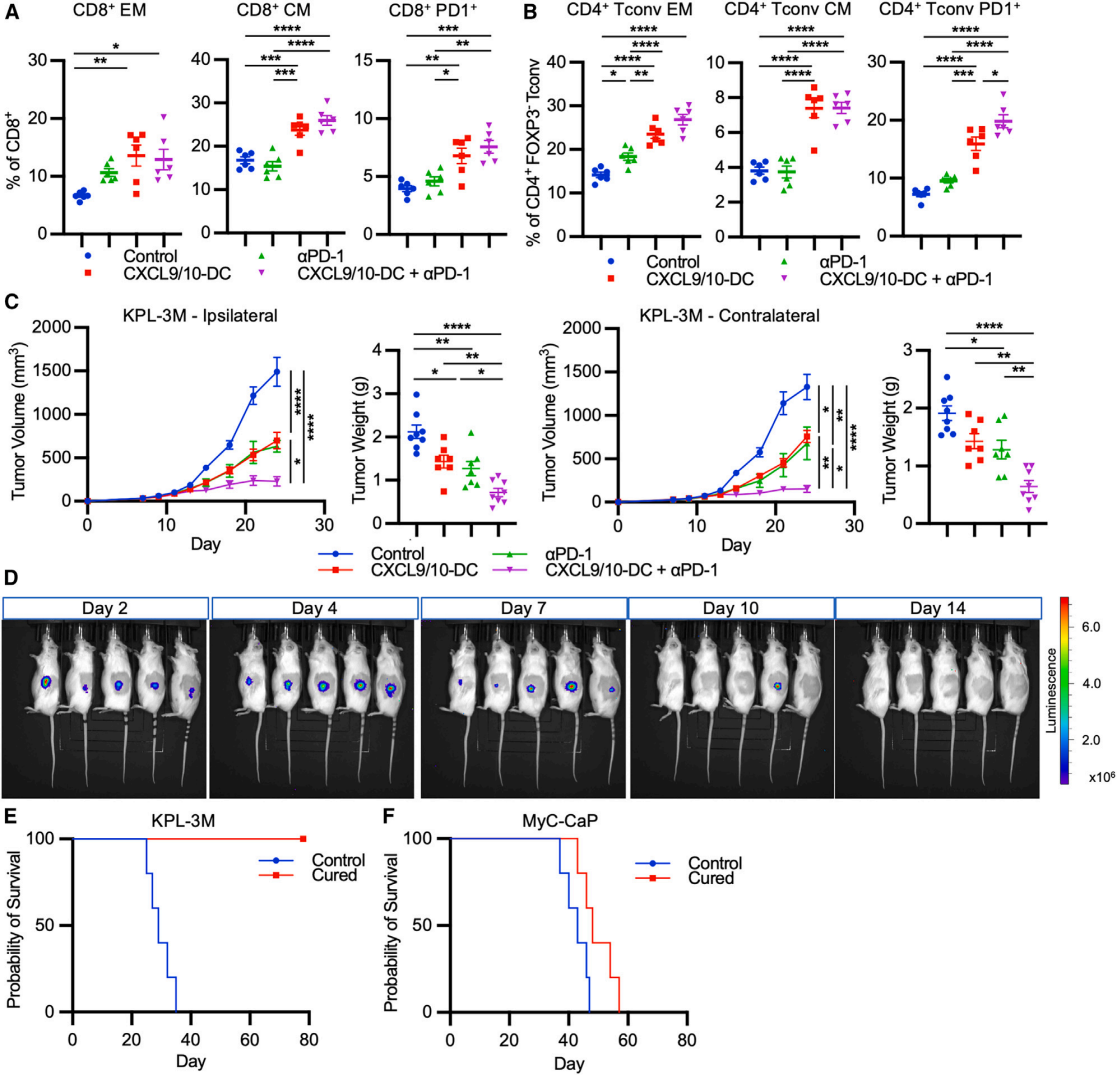
CXCL9/10-DC and anti-PD-1 combination treatment generates systemic tumor-specific immunity (A and B) Therapy-induced systemic changes in (A) CD8^+^ or (B) CD4^+^ T cells revealed by flow phenotyping. Spleens were collected on D16 post-inoculation (1.25 × 10^5^ KPL-3M delivered s.c. in FVB mice) following treatment with PBS control; CXCL9/10-DC (IT 1 × 10^6^ CXCL9-DC and CXCL10-DC each per injection on D6, D8, and D11); anti-PD-1 (i.p. injections at 200 μg/injection on D6, D8, D11, and D14); or the combination of CXCL9/10-DC and anti-PD-1 (n = 6–8 mice per group). Single-cell suspensions were prepared and subjected to flow cytometry. CM, CD44^+^CD62L^+^; EM, CD44^+^CD62L^−^. (C) FVB mice were inoculated bilaterally with 1.25 × 10^5^ KPL-3M cells. On D6, mice bearing ∼50-mm^3^ tumors were randomized and treated ipsilaterally with PBS control; CXCL9/10-DC (IT 1 × 10^6^ CXCL9-DC and CXCL10-DC each per injection on D6, D8, and D11); anti-PD-1 (i.p. injections at 200 μg/injection on D6, D8, D11, and D14); or the combination of CXCL9/10-DC and anti-PD-1 (n = 7–9 mice per group). Tumor growth curves and tumor weights at the time of euthanasia from treated (ipsilateral) and untreated (contralateral) tumors are presented. (D) CXCL9/10-DC and anti-PD-1 combination treatment generates systemic tumor-specific immune memory. FVB mice cured of KPL-3M tumors following combination therapy were inoculated with 2.5 × 10^5^ KPL-3M tumor cells on the contralateral side, 90 days after tumor rejection (n = 5 mice per group). *In vivo* bioluminescence imaging was used to monitor tumor growth through the luciferase reporter genetically engineered in the KPL-3M cells. (E and F) The specificity of systemic antitumor immune memory shown as a Kaplan-Meier plot of (E) KPL-3M cells or (F) syngeneic MyC-CaP cells. Error bars represent SEM. p values were determined by one-way ANOVA, adjusting for multiple comparisons for (A–C). ∗p < 0.05; ∗∗p < 0.005; ∗∗∗p < 0.0005; ∗∗∗∗p < 0.00005.

To assess systemic immune responses, spleens of KPL-3M tumor-bearing mice from the same studies as in Figure 6A were analyzed by flow cytometry. Whereas anti-PD-1 alone led to a modest increase in CD44^+^CD62L^−^ effector memory (EM) CD8^+^ and CD4^+^ T cells, CXCL9/10-DC treatment induced a significant increase in both EM and CD44^+^CD62L^+^ central memory (CM) T cells with a concurrent decrease in naive T cells (Figures 7A, 7B, S7A, and S7B). An increase in PD1^+^CD8^+^ T cells, which has been shown to contain a pool of systemic tumor-specific T cells,^42^^,^^43^ and PD1^+^CD4^+^ T cells was also observed in response to CXCL9/10-DC, with the highest magnitude following combination therapy (Figures 7A and 7B). To functionally assess potential systemic antitumor effects, mice bearing bilateral KPL-3M tumors were treated ipsilaterally with IT CXCL9/10 in combination with i.p. anti-PD-1, and bilateral tumor growth was monitored (Figure 7C). Ipsilateral tumor vaccination with IT CXCL9/10-DC resulted in the inhibition of contralateral tumor growth, suggestive of systemic immunity following local tumor vaccination. Addition of anti-PD-1 significantly augmented the efficacy of ipsilateral IT CXCL9/10-DC vaccination, indicative of improved systemic antitumor immune responses (Figure 7C).

To evaluate whether combination therapy led to the development of tumor-specific immune memory, tumor rechallenge was performed using mice that had eradicated KPL-3M tumors following CXCL9/10-DC combined with anti-PD-1. Cured mice and age-matched naive control mice were inoculated with KPL-3M tumor cells on the contralateral flank 3 months after the initial rejection of the primary tumors (Figure 7D). Tumor establishment was verified on D2 by bioluminescence imaging (Figure 7D). Tumor growth in cured mice continued to increase on D4 but decreased by D7 and was completely eradicated by D14 (Figures 7D and 7E). In contrast, all of the age-matched naive mice succumbed to tumor challenge in ∼30 days (Figure 7E). To confirm tumor specificity, cured mice were rechallenged with a syngeneic prostate cancer cell line, MyC-CaP (Figure 7F). Both naive and cured mice succumbed to the MyC-CaP tumor challenge, indicating that systemic antitumor immunity is specific to the KPL-3M tumors. These data demonstrate that the combination treatment with CXCL9/10-DC and anti-PD-1 leads to enduring systemic tumor-specific immune memory.

## Discussion

Despite recent progress in immunotherapy, the efficacy of ICB is constrained by therapeutic resistance in many patients with cancer.^5^ Two critical immune evasion mechanisms, impaired tumor antigen presentation and an immunosuppressive TME, pose challenges to generating effective antitumor T cell responses and drive resistance to ICB, particularly in patients with NSCLC.^5^^,^^44^^,^^45^^,^^46^^,^^47^ In this report, we demonstrate that *in situ* vaccination with CXCL9/10-DC elicits antitumor responses in multiple murine models of NSCLC and enhances the efficacy of PD-1/PD-L1 blockade. The efficacy of CXCL9/10-DC is dependent on both endogenous CD4^+^ and CD8^+^ T cells and partially dependent on the CXCR3 axis. Although CXCL9/10-DC monotherapy promotes T cell infiltration to the tumor margin, combination therapy with anti-PD-1 enhances T cell infiltration into the tumor and augments their proliferation and activity, resulting in the establishment of tumor-specific immunity. These findings underscore the potential of IT CXCL9/10-DC as a promising strategy for augmenting the efficacy of ICB in NSCLC.

Analysis of gene expression from the TCGA dataset revealed that the CXCL9/10 signature positively correlated with activated DC and M1 macrophages. Recent studies have identified tumor-infiltrating cDC1s and macrophages as critical sources of CXCR3 ligands necessary for sustained antitumor responses.^14^^,^^16^^,^^17^ High CXCL9/10 expression in the TCGA dataset also positively correlated with effector T cell signatures, consistent with the established function of CXCL9/10 in mediating chemotaxis of CXCR3^+^ effector T cells.^17^^,^^19^ However, not all tumors with high CXCL9/10 expression had high T cell infiltration (Figure S1A), suggesting alternative mechanisms of T cell exclusion.^5^^,^^48^^,^^49^ Although our analysis of gene signatures from TCGA highlights the association of the CXCL9/CXCL10/CXCR3 axis with adaptive immunity in the TME of NSCLC, results should be considered in the context of the limitations of this dataset. NSCLC in the TCGA dataset represents early-stage disease, amenable for surgical resection. In addition, evaluation of the TCGA database may underrepresent immune cells in the TME because of the requirements for high tumor contents in represented biorepositories.^50^

*In situ* vaccination with CXCL9/10-DC provided broad antitumor efficacy in multiple murine models of lung cancer and augmented the effectiveness of anti-PD-1 therapy in murine models of LKB1-deficient NSCLC. Spatial analysis of the KPL-3M tumors demonstrated an immunosuppressed TME with a paucity of T cells, consistent with previous findings in LKB1-deficient NSCLC.^32^^,^^33^^,^^34^ CXCL9/10-DC vaccination induced an initial recruitment of effector T cells, predominantly at the leading tumor edge (Figure 5), suggesting that tumor killing may be initiated at the tumor leading edge. The frequency of proliferating CD4^+^ and CD8^+^ T cells as well as GzmB^+^ CD8^+^ T cells declined over time, possibly due to inhibitory mechanisms within the TME. Addition of anti-PD-1 to IT CXCL9/10-DC therapy augmented the recruitment and infiltration of both CD4^+^ Tconv and GzmB^+^ CD8^+^ T cells into the tumors and sustained their proliferation, consistent with the enhanced efficacy of this combination therapy. In-depth subtyping of tumor-infiltrating T cells by scRNA-seq revealed a decrease in Tregs and a concurrent increase in effector CD8^+^ and CD4^+^ phenotypes, including both Th1 and Th2 subtypes. This finding aligns with our analysis of TCGA data, indicating a direct correlation between the CXCL9/10 signature and both Th1 and Th2 CD4^+^ T cells in LUAD and LUSC. Th1 cells express CXCR3, and lack of CXCR3 expression on Th2 cells suggests that the enrichment of these cells in the tumor is possibly due to an indirect effect of CXCL9/10 that alters the soluble milieu of the TME.^31^^,^^51^^,^^52^ Future studies are warranted to evaluate the functional importance of the Th2 subtype in CXCL9/10-DC-mediated antitumor responses.

*In vivo* DC trafficking studies reveal that IT CXCL9/10-DC vaccines, although short-lived, increased the levels of CXCL9/10 within the TME and induced rapid infiltration of endogenous DCs and T cells into the tumor (Figure S3). This observation is consistent with the recent finding that BMDC vaccines require host cDC1s for their antitumor effect.^38^ Approaches that can prolong DC survival *in vivo* should enhance the efficacy of *in situ* DC vaccination. A recent study showed that *Bcl2l1*, encoding the antiapoptotic protein BCLxL, is induced by CD40 signaling activation in cDC1 and that ectopic expression of BCLxL is sufficient to promote cDC1 survival in the absence of the endogenous CD40-CD40L signaling pathway.^53^ The contribution of DC in CXCL9/10-DC-induced antitumor effects via enhanced tumor antigen presentation and broader tumor-specific T cell activation will be evaluated in future studies, including therapy-induced changes in T cell receptor (TCR) clonality by longitudinal TCR sequencing as well as tumor immunoediting by whole exosome sequencing, as previously described.^54^

A recent meta-analysis of tumors from >1,000 patients revealed that CXCL9 expression was the second strongest predictor of response to ICB.^20^ The strong association between CXCL9/10 and effector T cell signatures observed in our NSCLC TCGA analysis as well as other studies suggests that the positive predictive value of CXCL9 as a biomarker of response to ICB could be due to its direct correlation with effector T cell responses, which are the critical immune mediators of response to immunotherapy.^19^^,^^55^^,^^56^ In accordance with this hypothesis, a recent study identified that homozygous loss at the 21.3 locus of the 9p chromosome (9p21) is associated with reduced CXCL9, as well as decreased cytotoxic CD8^+^ T cell number and TCR diversity in the TME, and drives resistance to ICB across multiple cancers.^57^^,^^58^^,^^59^ These results, in conjunction with our preclinical findings in this report, suggest that IT CXCL9/10 may facilitate antitumor T cell responses to overcome resistance to immunotherapy. This therapeutic approach could be particularly advantageous for patients with loss of the chromosome 9p21 locus, present in ∼15% of all human cancers, as well as other patients whose tumors demonstrate a paucity of T cell infiltration.^60^

We have previously demonstrated the clinical safety and feasibility of *in situ* vaccination with autologous chemokine gene-engineered DCs in patients with advanced NSCLC alone^61^ or in combination with ICB (this study was registered at ClinicalTrials.gov [NCT03546361]). The current preclinical findings of *in situ* vaccination with CXCL9/10-DC support the clinical investigation of this approach to enhance the efficacy of ICB in NSCLC.

### Limitations of the study

Our preclinical findings require consideration of the limitations of the study. Syngeneic murine models of NSCLC using subcutaneous (s.c.) tumor implantation lack a latency period and fail to replicate the intricate spectrum of lung tumorigenesis, ranging from premalignancy to invasive cancer. Moreover, the immune contexture of the TME in s.c.-implanted tumors may differ from that of the lung microenvironment. Technical challenges associated with frequent IT injections in orthotopic models of NSCLC limit the feasibility of evaluating *in situ* CXCL9/10-DC vaccination in lung tumor models.

## STAR★Methods

### Key resources table

**Table:** 

REAGENT or RESOURCE	SOURCE	IDENTIFIER
**Antibodies**		

CD45 PerCP/Cy5.5 Clone 30-F11	Biolegend	Cat #103132; RRID:AB_893340
CD11b AF700 Clone M1/70	Biolegend	Cat #101222; RRID:AB_493705
CD11c BV605 Clone N418	Biolegend	Cat #117334; RRID:AB_2562415
IA/IE BV650 Clone M5/114.15.2	Biolegend	Cat #107641; RRID:AB_2565975
XCR PE Clone ZET	Biolegend	Cat #148204; RRID:AB_2563843
CD103 APC Clone 2E7	Biolegend	Cat #121414; RRID:AB_1227502
Ly6C BV711 Clone HK1.4	Biolegend	Cat #128037; RRID:AB_2562630
Ly6G BV421 Clone 1A8	Biolegend	Cat #127628; RRID:AB_2562567
CD64 PE/Cy7 Clone X54-5/7.1	Biolegend	Cat #139314; RRID:AB_2563904
CD86 PE Clone A17199A	Biolegend	Cat #159203; RRID:AB_2832567
CD3 AF700 Clone 17A2	Biolegend	Cat #100216; RRID:AB_493697
CD4 BV650 Clone RM4-5	Biolegend	Cat #100555; RRID:AB_2562529
CD8 BV421 Clone 53-6.7	BD Biosciences	Cat #563898; RRID:AB_2738474
CD44 PE-Dazzle594 Clone IM7	Biolegend	Cat #103056; RRID:AB_2564044
CD62L APC Clone MEL-14	Biolegend	Cat #104412; RRID:AB_313099
CD25 BV711 Clone PC61	Biolegend	Cat #102049; RRID:AB_2564130
PD1 PE/Cy7 Clone 29F.1A12	Biolegend	Cat #135216; RRID:AB_10689635
Tim3 PE-Dazzle594 Clone B8.2C12	Biolegend	Cat #134014; RRID:AB_2632738
TNFα FITC Clone MP6-XT22	BD Biosciences	Cat #554418; RRID:AB_395379
IFNγ PE Clone XMG1.2	eBioscience	Cat #12-7311-82; RRID:AB_466193
IL-4 PE/Cy7 Clone 11B11	Biolegend	Cat #504118; RRID:AB_10898116
FoxP3 PE Clone 150D	Biolegend	Cat #320008; RRID:AB_492980
Ki67 BV605 Clone 16A8	Biolegend	Cat #652413; RRID:AB_2562664
CXCR3 FITC Clone CXCR3-173	Biolegend	Cat #126536; RRID:AB_2566565
41BB APC Clone 17B5	Biolegend	Cat #106110; RRID:AB_2564297
CD49b APC Clone HMα2	Biolegend	Cat #103515; RRID:AB_2566100
Zombie NIR	Biolegend	Cat #423105
CD4 Clone 4SM95	eBioscience	Cat #14-9766-82; RRID:AB_2573008
CD8 Clone 4SM15	eBiosciences	Cat #14-0808-82; RRID:AB_2572861
Granzyme B Clone E5V2L	Cell Signaling Technology	Cat #44153; RRID:AB_2857976
FoxP3 Clone D6O8R	Cell Signaling Technology	Cat #12653; RRID:AB_2797979
Ki67 Polyclonal Antibody	Bethyl Laboratories	Cat #IHC-00375; RRID:AB_1547959
PanCK Clone AE1/AE3/PCK26	Roche	Cat #760-2135; RRID:AB_2810237
PD-1 Clone RMP1-14	BioXCell	Cat #BE0146; RRID:AB_10949053
PD-L1 Clone 10F.9G2	BioXCell	Cat #BE0101; RRID:AB_10949073
CD8 Clone 2.43	BioXCell	Cat #BE0061; RRID:AB_1125541
CD4 Clone GK1.5	BioXCell	Cat #BE0003-1; RRID:AB_1107636
CXCR3 Clone CXCR3-173	BioXCell	Cat #BE0249; RRID:AB_2687730
Rat IgG2a isotype control	BioXCell	Cat #BE0089; RRID:AB_1107769
Rat IgG2b isotype control	BioXCell	Cat #BE0090; RRID:AB_1107780
OmniMap Anti-Rat HRP	Roche	Cat #760-4457; RRID:AB_3095527
OmniMap Anti-Mouse HRP	Roche	Cat #760-4310; RRID:AB_2885182
OmniMap Anti-Rabbit HRP	Roche	Cat #760-4311; RRID:AB_2811043
Collagenase IV	Roche	Cat #10103586001
Lysis Buffer	ThermoFisher	Cat #78501
Protease Inhibitor	ThermoFisher	Cat #A32953

**Chemicals, peptides, and recombinant proteins**		

Recombinant Murine GM-CSF	Peprotech	Cat #315-03
Recombinant Murine IL-4	Peprotech	Cat #214-14
Fingolimod hydrochloride	Cayman Chemical	Cat #10006292
Zinc fixative	BD Pharmingen	Cat # 552658

**Critical commercial assays**		

Celltracker Red CMTPX Dye	Invitrogen	Cat #C34552
FITC-Dextran	Sigma	Cat #FD40S100MG
Mouse CXCL9/MIG DuoSet ELISA	R&D Systems	Cat #DY492
Mouse CXCL10/IP-10/CRG-2 DuoSet ELISA	R&D Systems	Cat #DY466
Cell Stimulation Cocktail	eBioscience	Cat #00-4970-93
FOXP3 Transcription Factor Staining Buffer Set	eBioscience	Cat #00-5523-00
Intracellular (IC) Fixation Buffer	eBioscience	Cat #00-8222-49
Bright-Glo Luciferase Assay System	Promega	Cat #E2610
BCA Protein Assay	ThermoFisher	Cat #23225
Benchmark ULTRA CC1	Roche/Ventana	Cat #950-224
Benchmark ULTRA CC2	Roche/Ventana	Cat #950-223
Reaction Buffer Concentrate (10X)	Roche/Ventana	Cat #950-300
DISCOVERY Wash	Roche/Ventana	Cat #950-510
DISCOVERY Inhibitor	Roche/Ventana	Cat #760-4840
Opal 480	Akoya Biosciences	Cat #FP1500001KT
Opal 520	Akoya Biosciences	Cat #FP1487001KT
Opal 570	Akoya Biosciences	Cat #FP1488001KT
Opal 620	Akoya Biosciences	Cat #FP1495001KT
Opal 690	Akoya Biosciences	Cat #FP1497001KT
Opal 780	Akoya Biosciences	Cat #FP1501001KT
Spectral DAPI	Akoya Biosciences	Cat #FP1490
ProLong Diamond Antifade Mountant	Molecular Probes	Cat #P36970

**Deposited data**		

TCGA Data	UCSC Xena	UCSC XENA: https://xenabrowser.net/datapages/?hub=https://tcga.xenahubs.net:443
Gene expression data of murine tumors after CXCL9/10-DC treatment	This paper	GEO: GSE232730

**Experimental models: Cell lines**		

1940A-KPL (*Kras*^*G12D*^*Tp53*^*−/−*^*Lkb1*^*−/−*^*Luc)*	Steven Dubinett Laboratory	N/A
KPL-3M (*Kras*^*G12D*^*Tp53*^*−/−*^*Lkb1*^*−/−*^*Luc)*	Steven Dubinett Laboratory	N/A
KP-3M (*Kras*^*G12D*^*Tp53*^*−/−*^*)*	Steven Dubinett Laboratory	N/A
L1C2	Steven Dubinett Laboratory	N/A
LKR-13 (*Kras*^*G12D*^*)*	Jonathan Kurie Laboratory	N/A
MyC-CaP	ATCC	Cat #CRL-3255; RRID:CVCL_J703

**Experimental models: Organisms/strains**		

FVB/NCrl	Charles River Laboratories	RRID:IMSR_CRL:207
129-Elite	Charles River Laboratories	RRID:IMSR_CRL:476
BALB/c	Charles River Laboratories	RRID:IMSR_CRL:476

**Recombinant DNA**		

Plasmid: pHIV-CXCL9	This paper	N/A
Plasmid: pHIV-CXCL10	This paper	N/A
Plasmid: pHIV-EGFP	Addgene	Cat # 21373; RRID:Addgene_21373
Plasmid: pHIV-dTomato	Addgene	Cat # 21374; RRID:Addgene_21374

**Software and algorithms**		

Prism 9	GraphPad	https://www.graphpad.com/
FlowJo 10.9.0	BD Biosciences	https://www.flowjo.com/
NovoExpress 1.5.6	Agilent Technologies	https://www.agilent.com/
R 4.1.0	N/A	https://www.r-project.org/
CellRanger 4.0	10X Genomics	https://support.10xgenomics.com/single-cell-gene-expression/software
Seurat 3.1.5	N/A	https://satijalab.org/seurat
Panglao Database	N/A	https://panglaodb.se/
Xcell	N/A	http://timer.cistrome.org/
inForm	Akoya Biosciences	https://www.akoyabio.com/phenoimager/software/inform-tissue-finder/
HALO	Indica Labs	https://indicalab.com/halo/

### Resource availability

#### Lead contact

Further information and requests for resources and reagents should be directed to and will be fulfilled by the lead contact, Bin Liu (bliu@mednet.ucla.edu).

#### Materials availability

All unique/stable reagents generated in this study are available from the lead contact with a completed Materials Transfer Agreement.

#### Data and code availability

• Single-cell RNA-seq data have been deposited at GEO and are publicly available as of the date of publication. Accession number is listed in the key resource table. TCGA data were downloaded from the UCSC Xena Data Portal.
• This paper does not report original code. The STAR Methods provides the parameters utilized in the software algorithms.
• Any additional information required to reanalyze the data reported in this paper is available from the lead contact upon request.

### Experimental model and study participant details

#### *In vivo* studies

FVB/NCrl mice, 129-E mice and BALB/c were purchased from Charles River Laboratories. Tumor cells were s.c.-implanted in 7-9-week-old female mice at optimal doses as indicated in figure legends. Tumor length and width were measured by caliper and the volume calculated by the equation: 0.4∗length∗widthˆ2. For immunotherapy studies, mice bearing ∼50mm^3^ tumors were randomized and treated with genetically engineered DCs as detailed below, anti-PD-1 antibody (BioXcell, Clone RMP1-14), or anti-PD-L1 antibody (BioXcell, Clone 10F.9G2). In tumor re-challenge studies, mice were euthanized when tumor volume reached 1500 mm^3^. Mice were housed in pathogen-free facilities at UCLA and all procedures were approved by the UCLA Animal Research Committee (ARC protocol # 2017-049).

#### Cell lines

The murine cell lines 1940A, KPL-3M, KP-3M and LKR-13 have been described previously.^28^ L1C2 cells and MyC-CaP cells were acquired from American Type Culture Collection (ATCC). Cell lines were maintained in culture media (RPMI-1640 medium supplemented with 10% FBS and 1% penicillin/streptomycin) at 37°C in a humidified atmosphere of 5% CO_2_ and utilized before 5 passages. Cell cultures were routinely tested for mycoplasma contamination and genotyped to ensure authenticity and purity. All procedures have been approved by the UCLA Institutional Biosafety Committee (BUA-2019-233-012-A).

#### Human data

The NSCLC gene expression RNAseq data were downloaded from UCSC Xena for both TCGA Lung Adenocarcinoma (LUAD) and TCGA Lung Squamous Cell Carcinoma (LUSC). Immune profiles derived from RNA-seq using the Xcell approach were downloaded from TIMER 2. Spearman correlation coefficients (R basic package) were utilized to assess the association between abundance of immune cells and CXCL9/10 expression.

### Method details

#### Generation of CXCL9/10-DC

BMDCs were generated as previously described.^24^^,^^27^ Briefly, bone marrow cells were cultured in DC media: 10% FBS in RPMI 1640 with 20 ng/mL murine GM-CSF (Peprotech) and 10 ng/mL murine IL-4 (Peprotech). Cells were seeded at 3 x 10^6^ cells/mL into a sterile non-tissue culture-treated 6 well plastic plate (Falcon) at 2 mL per well in a humidified CO_2_ incubator (37°C, 5% CO_2_). On D3, media was changed with fresh DC media without depletion of loosely adherent cells. On D6, DCs were harvested by gently pipetting loosely adherent and floating cells. Cells were resuspended at a concentration of 1 x 10^6^ cells/mL into a new non-tissue culture-treated 6 well plastic plate and spinfected at 800 *g* for 2 h at 32°C in the presence of lentivirus encoding murine CXCL9 (pHIV-CXCL9) or CXCL10 (pHIV-CXCL10). Following transduction, cells were washed two times with DPBS (Corning) prior to injection. Equal numbers of cells transduced to secrete CXCL9 or CXCL10 were combined to constitute CXCL9/10-DC. DC viability was assessed by Trypan-blue staining before and after CXCL9/10 transduction for all experiments. For trafficking experiments, Mock-DCs and CXCL9/10-DCs were labeled with CellTracker Red CMTPX Dye (Invitrogen) per manufacturer’s protocol. Murine CXCL9 coding sequence was inserted into pHIV-EGFP (Addgene) using the cloning sites *XbaI* (NEB) and *BamHI* (NEB) to generate pHIV-CXCL9*.* For pHIV-CXCL10, murine CXCL10 coding sequence was inserted into pHIV-dTomato (Addgene) with *EcoRI* (NEB) and *XbaI* (NEB).

#### FITC-Dextran uptake

FITC-Dextran solution (Sigma) was made at a concentration of 25 mg/mL in Milli-Q water. FACS tubes were pre-conditioned at 4°C or 37°C and 2 x 10^6^ DCs were placed in 200 μL of RPMI +10% FBS. 10 μL of the FITC-Dextran solution was added to the tubes and placed either at 4°C or 37°C for 15 min. DCs without FITC-Dextran were incubated at 37°C for 15 min as a control. After 15 min, cells were washed with 1 mL of FACS Buffer (1X DPBS +2% FBS) and spun for 5 min at 1,500 RPM. After spinning, cells were then fixed with 100 μL of 0.5% Formalin (Epredia) for 20 min at room temperature. Cells were washed 2X with FACS buffer and analyzed by flow cytometry using the NovoCyte Quanteon Flow Cytometer (Agilent).

#### ELISA

Quantification of mouse CXCL9 (DY#492) and CXCL10 (DY#466) secretion from lentivirally transduced DCs or tumor extracts was performed using Mouse DuoSet ELISA Kit following the manufacturer’s directions (R&D Systems). The absorbance readings were taken using a BioRad absorbance plate reader. A standard curve was created by plotting the mean absorbance for each standard on the y axis against the concentration on the x axis and a best fit curve was drawn. Sample concentrations were determined based on the standard curve.

#### *In vivo* antibody-mediated cell depletion and T cell recruitment

CD8^+^ and CD4^+^ T cell depletions were accomplished with mouse anti-CD8 (BioXcell, Clone 2.43) and mouse anti-CD4 (BioXcell, Clone GK1.5), respectively. Depleting antibodies were given via i.p. injection at 200 μg/injection every two days starting on D6. For T cell recruitment studies, mouse anti-CXCR3 antibody (BioXcell, Clone CXCR3-173) was administered i.p, at 200 μg/injection. Prevention of T cell LN egress was performed with fingolimod hydrochloride (FTY720; Cayman, 10006292) administered i.p. every other day at 2 mg/kg. All antibody experiments utilized isotype controls (BioXcell, Clone 2A3 or LTF-2).

#### Tissue preparation for single cell suspension, ELISA, and metastatic cell growth

Single cell suspension of murine tumor, spleen and TDLN were performed as previously described^27^^,^^28^ and subjected to flow phenotyping and scRNA-seq analyses. For tumor extracts utilized in ELISA, a quarter of each tumor was homogenized for 6 s in 300 μL of cold lysis buffer (ThermoFisher) with protease inhibitor (ThermoFisher). Samples were incubated on ice for 20 min and subsequently stored at −80°C. Prior to performing ELISA, samples were thawed and centrifuged at 14,000 g for 15 min. Equal amounts of protein from each sample, as determined by BCA Protein Assay (ThermoFisher), were utilized for ELISA. To prepare lung tissues for the evaluation of metastatic tumor cell growth, murine lungs were harvested and minced into smaller pieces using a sharp blade before incubation with 1 mg/mL Collagenase IV (Roche) for 1 h at 37°C with frequent agitation. Following red blood cell (RBC) lysis, cells were filtered through a 100μm filter and plated in a 6-well plate. At 24 h, non-adherent cells were removed. On D10, tumor cells were quantified by luminescence using the Bright-Glo Luciferase Assay System (Promega).

#### Immunophenotyping by flow cytometry

Surface staining of single cell suspensions was performed for 30 min at 4°C as previously described.^27^^,^^28^ Intracellular staining for FOXP3 and Ki67 was performed using the FOXP3 Transcription Factor Staining Buffer Set (eBioscience) per manufacturer’s protocol. Cytokine production was evaluated by intracellular staining after *in vitro* stimulation with Cell Stimulation Cocktail (eBioscience) for 4 h, using the intracellular fixation buffer (eBioscience), as previously described.^28^ CXCR3 staining in the tumor was also performed using the intracellular fixation buffer (eBioscience), given that it has been demonstrated in the literature that CXCR3 receptor can be internalized upon ligand binding.^62^^,^^63^ Data acquisition was performed on Attune NxT cytometer (ThermoFisher) and Novocyte Quanteon (Agilent), and data analyzed by FlowJo software (TreeStar).

#### scRNA-seq following flow sorting

Single cell suspensions from 5 murine tumors in each treatment group were pooled, stained with Zombie-NIR LIVE/DEAD stain and CD45 antibody for 30 min at 4°C. Live/CD45^+^ leukocytes were sorted by BD Biosciences Aria II cell sorter with 100μm nozzle. Samples were processed for 10X Single Cell 3′ Gene Expression V3 (10X Genomics) with Novaseq S2 (Illumina) with paired ends for library construction and sequencing performed at the UCLA Technology Center for Genomics & Bioinformatics (TCGB) core.

Raw reads were aligned by CellRanger 4.0 using *mm10* for the reference genome and *Ensembl* v.93 for transcript annotation. Raw count matrices were processed further by *DoubletFinder* to detect doublets and *Seurat* pipeline (v 3.1.5) for pre-processing, dimensional reduction, and cell clustering analyses. In brief, low-quality cells were filtered out if mitochondrial content >10%, detected genes <250, and total UMI > 10^5^. A total of 50,771 cells were retained for further analysis. The data was normalized using the Seurat *sctransform* approach for the top 3,000 highest variance genes.^64^ The Louvain algorithm-based cluster analyses were based on the first 35 principal components with a resolution of 1.2 and visualized by the uniform manifold approximation and projection (UMAP) method. Cell clusters were annotated by major lineages, using the enrichment approach to determine if the overlap between cluster and cell lineage markers based on Panglao database is significant. For further analysis, cells were re-clustered to determine subpopulations and trajectory based on pseudotime analysis using the R *slingshot* package.

#### Multiplex immunofluorescence (MIF)

Tumor tissues were collected and fixed with zinc fixative (BD Pharmingen) for 24–48 h before embedding.^65^ MIF was performed utilizing the Ventana Discovery Ultra (Roche) and Opal fluorophores (Akoya Biosciences). 5μm-thick tissue sections on Superfrost microscopic slides (VWR International) were deparaffinized using EZ-Prep reagent (Roche) followed by antigen retrieval in CC1 buffer (pH 9, 95°C; Roche). Discovery Inhibitor (Roche) was applied to inhibit enzymatic activities followed by 6 sequential rounds of staining. Each round included the addition of a primary antibody followed by detection using the OmniMap secondary antibody (Roche). Signal amplification was performed utilizing Opal fluorophores at the conditions suggested by the manufacturer. Between rounds of staining, the tissue sections underwent heat-induced epitope retrieval to remove the primary-secondary-HRP antibody complexes before staining with the subsequent antibody. The primary antibodies and corresponding fluorophores were stained with Opal 480, 520, 570, 620, 690 and 780 (Akoya Biosciences). The slides were then counterstained with Spectral DAPI (Akoya Biosciences) and mounted with ProLong Diamond antifade mounting medium (Thermo Fisher Scientific). Stained slides were imaged using the Vectra Polaris imaging system (Akoya Biosciences). A whole slide scan was acquired with 20x resolution and imported into the inForm software (Akoya Biosciences) followed by spectral unmixing. Slide scans were then imported into HALO (Indica Labs) for stitching and whole slide spatial analysis. Cell characterization and phenotyping were performed before spatial analysis of the whole tumor region. The data was then exported and graphed with Prism (Graphpad). The representative MIF images were exported using inForm software following spectral unmixing.

### Quantification and statistical analysis

Experiments were performed at least twice except for the scRNA-seq and MIF studies, which were performed once. Results from one representative experiment are shown with biological replicates detailed in figure legends. Statistical analyses were performed in Prism 9 (GraphPad) unless otherwise noted. Statistical significance was determined using an unpaired, parametric *t*-test with 95% confidence interval. Results are reported as mean ± SEM, unless indicated. Differences between groups were assessed using either two-tailed unpaired *t*-tests or one-way ANOVA with Bonferroni multiple-comparisons. Statistical significance is reported as the following: ∗p < 0.05; ∗∗p < 0.01; ∗∗∗p < 0.001; ∗∗∗∗p < 0.0001.

## Supplemental information

Document S1. Figures S1–S7

Document S2. Article plus supplemental information

## References

[1] Reck M., Rodríguez-Abreu D., Robinson A.G., Hui R., Csőszi T., Fülöp A., Gottfried M., Peled N., Tafreshi A., Cuffe S. (2016). Pembrolizumab versus Chemotherapy for PD-L1-Positive Non-Small-Cell Lung Cancer. N. Engl. J. Med..

[2] Gandhi L., Garassino M.C. (2018). Pembrolizumab plus Chemotherapy in Lung Cancer. N. Engl. J. Med..

[3] Garon E.B., Hellmann M.D., Rizvi N.A., Carcereny E., Leighl N.B., Ahn M.J., Eder J.P., Balmanoukian A.S., Aggarwal C., Horn L. (2019). Five-Year Overall Survival for Patients With Advanced NonSmall-Cell Lung Cancer Treated With Pembrolizumab: Results From the Phase I KEYNOTE-001 Study. J. Clin. Oncol..

[4] Garassino M.C., Gadgeel S., Speranza G., Felip E., Esteban E., Dómine M., Hochmair M.J., Powell S.F., Bischoff H.G., Peled N. (2023). Pembrolizumab Plus Pemetrexed and Platinum in Nonsquamous Non-Small-Cell Lung Cancer: 5-Year Outcomes From the Phase 3 KEYNOTE-189 Study. J. Clin. Oncol..

[5] Sharma P., Hu-Lieskovan S., Wargo J.A., Ribas A. (2017). Primary, Adaptive, and Acquired Resistance to Cancer Immunotherapy. Cell.

[6] Cortellini A., Cannita K., Tiseo M., Cortinovis D.L., Aerts J., Baldessari C., Giusti R., Ferrara M.G., D'Argento E., Grossi F. (2021). Post-progression outcomes of NSCLC patients with PD-L1 expression >/= 50% receiving first-line single-agent pembrolizumab in a large multicentre real-world study. Eur. J. Cancer.

[7] Insa A., Martín-Martorell P., Di Liello R., Fasano M., Martini G., Napolitano S., Vicidomini G., Cappabianca S., Franco R., Morgillo F., Della Corte C.M. (2022). Which treatment after first line therapy in NSCLC patients without genetic alterations in the era of immunotherapy?. Crit. Rev. Oncol. Hematol..

[8] Auclin E., Benitez-Montanez J., Tagliamento M., Parisi F., Gorria T., Garcia-Campelo R., Dempsey N., Pinato D.J., Reyes R., Albarrán-Artahona V. (2023). Second-line treatment outcomes after progression from first-line chemotherapy plus immunotherapy in patients with advanced non-small cell lung cancer. Lung Cancer.

[9] Kazandjian D., Keegan P., Suzman D.L., Pazdur R., Blumenthal G.M. (2017). Characterization of outcomes in patients with metastatic non-small cell lung cancer treated with programmed cell death protein 1 inhibitors past RECIST version 1.1-defined disease progression in clinical trials. Semin. Oncol..

[10] Herbst R.S., Soria J.C., Kowanetz M., Fine G.D., Hamid O., Gordon M.S., Sosman J.A., McDermott D.F., Powderly J.D., Gettinger S.N. (2014). Predictive correlates of response to the anti-PD-L1 antibody MPDL3280A in cancer patients. Nature.

[11] Tumeh P.C., Harview C.L., Yearley J.H., Shintaku I.P., Taylor E.J.M., Robert L., Chmielowski B., Spasic M., Henry G., Ciobanu V. (2014). PD-1 blockade induces responses by inhibiting adaptive immune resistance. Nature.

[12] Garon E.B., Rizvi N.A., Hui R., Leighl N., Balmanoukian A.S., Eder J.P., Patnaik A., Aggarwal C., Gubens M., Horn L. (2015). Pembrolizumab for the treatment of non-small-cell lung cancer. N. Engl. J. Med..

[13] Kohli K., Pillarisetty V.G., Kim T.S. (2022). Key chemokines direct migration of immune cells in solid tumors. Cancer Gene Ther..

[14] Spranger S., Dai D., Horton B., Gajewski T.F. (2017). Tumor-Residing Batf3 Dendritic Cells Are Required for Effector T Cell Trafficking and Adoptive T Cell Therapy. Cancer Cell.

[15] Pascual-García M., Bonfill-Teixidor E., Planas-Rigol E., Rubio-Perez C., Iurlaro R., Arias A., Cuartas I., Sala-Hojman A., Escudero L., Martínez-Ricarte F. (2019). LIF regulates CXCL9 in tumor-associated macrophages and prevents CD8(+) T cell tumor-infiltration impairing anti-PD1 therapy. Nat. Commun..

[16] House I.G., Savas P., Lai J., Chen A.X.Y., Oliver A.J., Teo Z.L., Todd K.L., Henderson M.A., Giuffrida L., Petley E.V. (2020). Macrophage-Derived CXCL9 and CXCL10 Are Required for Antitumor Immune Responses Following Immune Checkpoint Blockade. Clin. Cancer Res..

[17] Chow M.T., Ozga A.J., Servis R.L., Frederick D.T., Lo J.A., Fisher D.E., Freeman G.J., Boland G.M., Luster A.D. (2019). Intratumoral Activity of the CXCR3 Chemokine System Is Required for the Efficacy of Anti-PD-1 Therapy. Immunity.

[18] Tokunaga R., Zhang W., Naseem M., Puccini A., Berger M.D., Soni S., McSkane M., Baba H., Lenz H.J. (2018). CXCL9, CXCL10, CXCL11/CXCR3 axis for immune activation - A target for novel cancer therapy. Cancer Treat Rev..

[19] Hoch T., Schulz D., Eling N., Gómez J.M., Levesque M.P., Bodenmiller B. (2022). Multiplexed imaging mass cytometry of the chemokine milieus in melanoma characterizes features of the response to immunotherapy. Sci. Immunol..

[20] Litchfield K., Reading J.L., Puttick C., Thakkar K., Abbosh C., Bentham R., Watkins T.B.K., Rosenthal R., Biswas D., Rowan A. (2021). Meta-analysis of tumor- and T cell-intrinsic mechanisms of sensitization to checkpoint inhibition. Cell.

[21] Ayers M., Lunceford J., Nebozhyn M., Murphy E., Loboda A., Kaufman D.R., Albright A., Cheng J.D., Kang S.P., Shankaran V. (2017). IFN-gamma-related mRNA profile predicts clinical response to PD-1 blockade. J. Clin. Invest..

[22] Santos P.M., Butterfield L.H. (2018). Dendritic Cell-Based Cancer Vaccines. J. Immunol..

[23] Champiat S., Tselikas L., Farhane S., Raoult T., Texier M., Lanoy E., Massard C., Robert C., Ammari S., De Baère T., Marabelle A. (2021). Intratumoral Immunotherapy: From Trial Design to Clinical Practice. Clin. Cancer Res..

[24] Yang S.C., Hillinger S., Riedl K., Zhang L., Zhu L., Huang M., Atianzar K., Kuo B.Y., Gardner B., Batra R.K. (2004). Intratumoral administration of dendritic cells overexpressing CCL21 generates systemic antitumor responses and confers tumor immunity. Clin. Cancer Res..

[25] Miller P.W., Sharma S., Stolina M., Butterfield L.H., Luo J., Lin Y., Dohadwala M., Batra R.K., Wu L., Economou J.S., Dubinett S.M. (2000). Intratumoral administration of adenoviral interleukin 7 gene-modified dendritic cells augments specific antitumor immunity and achieves tumor eradication. Hum. Gene Ther..

[26] Kirk C.J., Hartigan-O'Connor D., Nickoloff B.J., Chamberlain J.S., Giedlin M., Aukerman L., Mule J.J. (2001). T cell-dependent antitumor immunity mediated by secondary lymphoid tissue chemokine: augmentation of dendritic cell-based immunotherapy. Cancer Res..

[27] Salehi-Rad R., Lim R.J., Du Y., Tran L.M., Li R., Ong S.L., Ling Huang Z., Dumitras C., Zhang T., Park S.J. (2023). CCL21-DC in situ vaccination in murine NSCLC overcomes resistance to immunotherapy and generates systemic tumor-specific immunity. J. Immunother. Cancer.

[28] Salehi-Rad R., Li R., Tran L.M., Lim R.J., Abascal J., Momcilovic M., Park S.J., Ong S.L., Shabihkhani M., Huang Z.L. (2021). Novel Kras-mutant murine models of non-small cell lung cancer possessing co-occurring oncogenic mutations and increased tumor mutational burden. Cancer Immunol. Immunother..

[29] Aran D., Hu Z., Butte A.J. (2017). xCell: digitally portraying the tissue cellular heterogeneity landscape. Genome Biol..

[30] Li T., Fu J., Zeng Z., Cohen D., Li J., Chen Q., Li B., Liu X.S. (2020). TIMER2.0 for analysis of tumor-infiltrating immune cells. Nucleic Acids Res..

[31] Muehlinghaus G., Cigliano L., Huehn S., Peddinghaus A., Leyendeckers H., Hauser A.E., Hiepe F., Radbruch A., Arce S., Manz R.A. (2005). Regulation of CXCR3 and CXCR4 expression during terminal differentiation of memory B cells into plasma cells. Blood.

[32] Skoulidis F., Goldberg M.E., Greenawalt D.M., Hellmann M.D., Awad M.M., Gainor J.F., Schrock A.B., Hartmaier R.J., Trabucco S.E., Gay L. (2018). STK11/LKB1 Mutations and PD-1 Inhibitor Resistance in KRAS-Mutant Lung Adenocarcinoma. Cancer Discov..

[33] Koyama S., Akbay E.A., Li Y.Y., Aref A.R., Skoulidis F., Herter-Sprie G.S., Buczkowski K.A., Liu Y., Awad M.M., Denning W.L. (2016). STK11/LKB1 Deficiency Promotes Neutrophil Recruitment and Proinflammatory Cytokine Production to Suppress T-cell Activity in the Lung Tumor Microenvironment. Cancer Res..

[34] Li R., Salehi-Rad R., Crosson W., Momcilovic M., Lim R.J., Ong S.L., Huang Z.L., Zhang T., Abascal J., Dumitras C. (2021). Inhibition of Granulocytic Myeloid-Derived Suppressor Cells Overcomes Resistance to Immune Checkpoint Inhibition in LKB1-Deficient Non-Small Cell Lung Cancer. Cancer Res..

[35] Sharma S., Yang S.C., Hillinger S., Zhu L.X., Huang M., Batra R.K., Lin J.F., Burdick M.D., Strieter R.M., Dubinett S.M. (2003). SLC/CCL21-mediated anti-tumor responses require IFNgamma, MIG/CXCL9 and IP-10/CXCL10. Mol. Cancer.

[36] Jin H.T., Anderson A.C., Tan W.G., West E.E., Ha S.J., Araki K., Freeman G.J., Kuchroo V.K., Ahmed R. (2010). Cooperation of Tim-3 and PD-1 in CD8 T-cell exhaustion during chronic viral infection. Proc. Natl. Acad. Sci. USA.

[37] Im S.J., Hashimoto M., Gerner M.Y., Lee J., Kissick H.T., Burger M.C., Shan Q., Hale J.S., Lee J., Nasti T.H. (2016). Defining CD8+ T cells that provide the proliferative burst after PD-1 therapy. Nature.

[38] Ferris S.T., Ohara R.A., Ou F., Wu R., Huang X., Kim S., Chen J., Liu T.T., Schreiber R.D., Murphy T.L., Murphy K.M. (2022). cDC1 Vaccines Drive Tumor Rejection by Direct Presentation Independently of Host cDC1. Cancer Immunol. Res..

[39] Yang H., Yamazaki T., Pietrocola F., Zhou H., Zitvogel L., Ma Y., Kroemer G. (2015). STAT3 Inhibition Enhances the Therapeutic Efficacy of Immunogenic Chemotherapy by Stimulating Type 1 Interferon Production by Cancer Cells. Cancer Res..

[40] Massi D., Rulli E., Cossa M., Valeri B., Rodolfo M., Merelli B., De Logu F., Nassini R., Del Vecchio M., Di Guardo L. (2019). The density and spatial tissue distribution of CD8(+) and CD163(+) immune cells predict response and outcome in melanoma patients receiving MAPK inhibitors. J. Immunother. Cancer.

[41] Sudmeier L.J., Hoang K.B., Nduom E.K., Wieland A., Neill S.G., Schniederjan M.J., Ramalingam S.S., Olson J.J., Ahmed R., Hudson W.H. (2022). Distinct phenotypic states and spatial distribution of CD8(+) T cell clonotypes in human brain metastases. Cell Rep. Med..

[42] Chen Z., Ji Z., Ngiow S.F., Manne S., Cai Z., Huang A.C., Johnson J., Staupe R.P., Bengsch B., Xu C. (2019). TCF-1-Centered Transcriptional Network Drives an Effector versus Exhausted CD8 T Cell-Fate Decision. Immunity.

[43] Kamphorst A.O., Pillai R.N., Yang S., Nasti T.H., Akondy R.S., Wieland A., Sica G.L., Yu K., Koenig L., Patel N.T. (2017). Proliferation of PD-1+ CD8 T cells in peripheral blood after PD-1-targeted therapy in lung cancer patients. Proc. Natl. Acad. Sci. USA.

[44] Cummings A.L., Gukasyan J., Lu H.Y., Grogan T., Sunga G., Fares C.M., Hornstein N., Zaretsky J., Carroll J., Bachrach B. (2020). Mutational landscape influences immunotherapy outcomes among patients with non-small-cell lung cancer with human leukocyte antigen supertype B44. Nat. Can. (Ott.).

[45] McGranahan N., Rosenthal R., Hiley C.T., Rowan A.J., Watkins T.B.K., Wilson G.A., Birkbak N.J., Veeriah S., Van Loo P., Herrero J. (2017). Allele-Specific HLA Loss and Immune Escape in Lung Cancer Evolution. Cell.

[46] Marty R., Kaabinejadian S., Rossell D., Slifker M.J., van de Haar J., Engin H.B., de Prisco N., Ideker T., Hildebrand W.H., Font-Burgada J., Carter H. (2017). MHC-I Genotype Restricts the Oncogenic Mutational Landscape. Cell.

[47] Salehi-Rad R., Li R., Paul M.K., Dubinett S.M., Liu B. (2020). The Biology of Lung Cancer: Development of More Effective Methods for Prevention, Diagnosis, and Treatment. Clin. Chest Med..

[48] Karin N. (2020). CXCR3 Ligands in Cancer and Autoimmunity, Chemoattraction of Effector T Cells, and Beyond. Front. Immunol..

[49] Bronger H., Singer J., Windmüller C., Reuning U., Zech D., Delbridge C., Dorn J., Kiechle M., Schmalfeldt B., Schmitt M., Avril S. (2016). CXCL9 and CXCL10 predict survival and are regulated by cyclooxygenase inhibition in advanced serous ovarian cancer. Br. J. Cancer.

[50] Cancer Genome Atlas Research Network (2014). Comprehensive molecular profiling of lung adenocarcinoma. Nature.

[51] Nagarsheth N., Wicha M.S., Zou W. (2017). Chemokines in the cancer microenvironment and their relevance in cancer immunotherapy. Nat. Rev. Immunol..

[52] Bonecchi R., Bianchi G., Bordignon P.P., D'Ambrosio D., Lang R., Borsatti A., Sozzani S., Allavena P., Gray P.A., Mantovani A., Sinigaglia F. (1998). Differential expression of chemokine receptors and chemotactic responsiveness of type 1 T helper cells (Th1s) and Th2s. J. Exp. Med..

[53] Wu R., Ohara R.A., Jo S., Liu T.T., Ferris S.T., Ou F., Kim S., Theisen D.J., Anderson D.A., Wong B.W. (2022). Mechanisms of CD40-dependent cDC1 licensing beyond costimulation. Nat. Immunol..

[54] Krysan K., Tran L.M., Grimes B.S., Fishbein G.A., Seki A., Gardner B.K., Walser T.C., Salehi-Rad R., Yanagawa J., Lee J.M. (2019). The immune contexture associates with the genomic landscape in lung adenomatous premalignancy. Cancer Res..

[55] Zumwalt T.J., Arnold M., Goel A., Boland C.R. (2015). Active secretion of CXCL10 and CCL5 from colorectal cancer microenvironments associates with GranzymeB+ CD8+ T-cell infiltration. Oncotarget.

[56] Reschke R., Gajewski T.F. (2022). CXCL9 and CXCL10 bring the heat to tumors. Sci. Immunol..

[57] Han G., Yang G., Hao D., Lu Y., Thein K., Simpson B.S., Chen J., Sun R., Alhalabi O., Wang R. (2021). 9p21 loss confers a cold tumor immune microenvironment and primary resistance to immune checkpoint therapy. Nat. Commun..

[58] Davoli T., Uno H., Wooten E.C., Elledge S.J. (2017). Tumor aneuploidy correlates with markers of immune evasion and with reduced response to immunotherapy. Science.

[59] William W.N., Zhao X., Bianchi J.J., Lin H.Y., Cheng P., Lee J.J., Carter H., Alexandrov L.B., Abraham J.P., Spetzler D.B. (2021). Immune evasion in HPV(-) head and neck precancer-cancer transition is driven by an aneuploid switch involving chromosome 9p loss. Proc. Natl. Acad. Sci. USA.

[60] Beroukhim R., Mermel C.H., Porter D., Wei G., Raychaudhuri S., Donovan J., Barretina J., Boehm J.S., Dobson J., Urashima M. (2010). The landscape of somatic copy-number alteration across human cancers. Nature.

[61] Lee J.M., Lee M.H., Garon E., Goldman J.W., Salehi-Rad R., Baratelli F.E., Schaue D., Wang G., Rosen F., Yanagawa J. (2017). Phase I Trial of Intratumoral Injection of CCL21 Gene-Modified Dendritic Cells in Lung Cancer Elicits Tumor-Specific Immune Responses and CD8+ T-cell Infiltration. Clin. Cancer Res..

[62] Colvin R.A., Campanella G.S.V., Sun J., Luster A.D. (2004). Intracellular domains of CXCR3 that mediate CXCL9, CXCL10, and CXCL11 function. J. Biol. Chem..

[63] Eiger D.S., Boldizsar N., Honeycutt C.C., Gardner J., Kirchner S., Hicks C., Choi I., Pham U., Zheng K., Warman A. (2022). Location bias contributes to functionally selective responses of biased CXCR3 agonists. Nat. Commun..

[64] Hafemeister C., Satija R. (2019). Normalization and variance stabilization of single-cell RNA-seq data using regularized negative binomial regression. Genome Biol..

[65] Mori H., Soonsawad P., Schuetter L., Chen Q., Hubbard N.E., Cardiff R.D., Borowsky A.D. (2015). Introduction of Zinc-salt Fixation for Effective Detection of Immune Cell-related Markers by Immunohistochemistry. Toxicol. Pathol..

