# Supplementary material for: CXCL9/10-engineered dendritic cells promote T cell activation and enhance immune checkpoint blockade for lung cancer
Source: Cell Rep Med. 2024 Mar 21;5(4):101479. doi: 10.1016/j.xcrm.2024.101479 (PMC11031384; doi:10.1016/j.xcrm.2024.101479)
Supplement: Document S1. Figures S1–S7 [file mmc1.pdf]

Cell Reports Medicine, Volume *5*

Supplemental information

CXCL9/10-engineered dendritic cells promote T cell activation and enhance immune checkpoint blockade for lung cancer

Raymond J. Lim, Ramin Salehi-Rad, Linh M. Tran, Michael S. Oh, Camelia Dumitras, William P. Crosson, Rui Li, Tejas S. Patel, Samantha Man, Cara E. Yean, Jensen Abascal, ZiLing Huang, Stephanie L. Ong, Kostyantyn Krysan, Steven M. Dubinett, and Bin Liu

SUPPLEMENTAL INFORMATION

CXCL9/10-engineered dendritic cells promote T cell activation and enhance immune checkpoint blockade for lung cancer

Raymond J. Lim, Ramin Salehi-Rad, Linh M. Tran, Michael S. Oh, Camelia Dumitras, William P. Crosson, Rui Li, Tejas S. Patel, Samantha Man, Cara E. Yean, Jensen Abascal, ZiLing Huang, Stephanie L. Ong, Kostyantyn Krysan, Steven M. Dubinett, Bin Liu

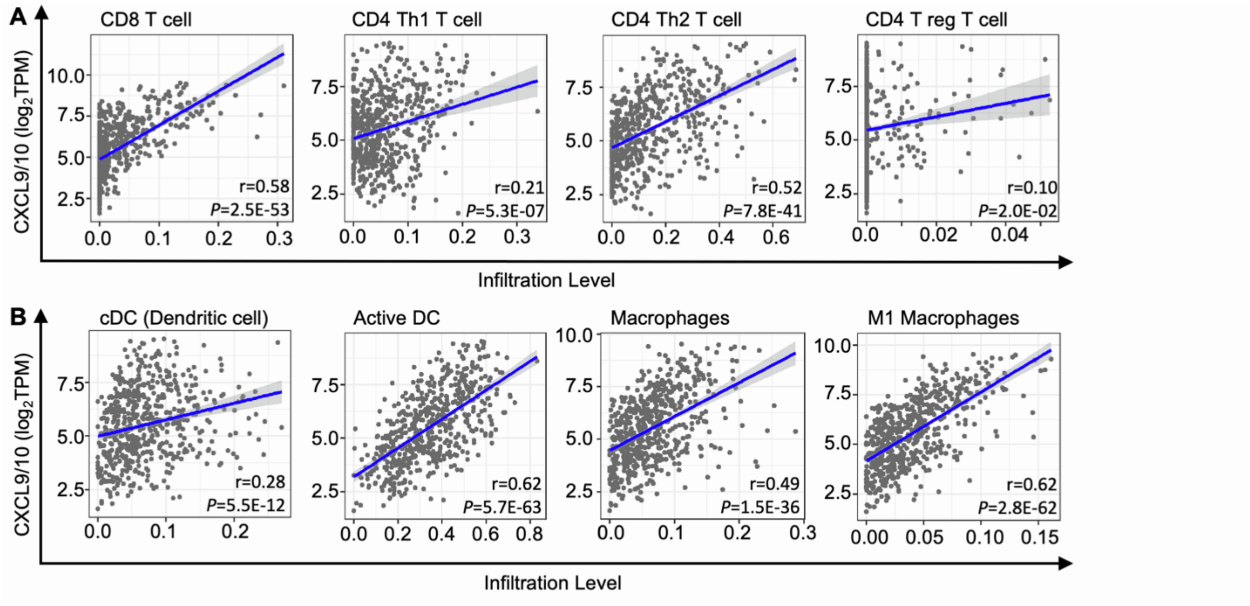

Figure S1. The Spearman correlations graphs between CXCL9/10 signature and immune cell infiltration derived from the TCGA database in LUAD. Related to Figure 1.

(**A**) $CD8^+$ T cells and $CD4^+$ Th2 T cells, but not Treg cells, positively correlate with CXCL9/10 signature levels. A weak correlation was found in $CD4^+$ Th1 T cells.

(**B**) CXCL9/10 expression levels positively correlate with activated dendritic cells, total macrophages and M1 subtype macrophages. A weaker correlation was found with total dendritic cells.

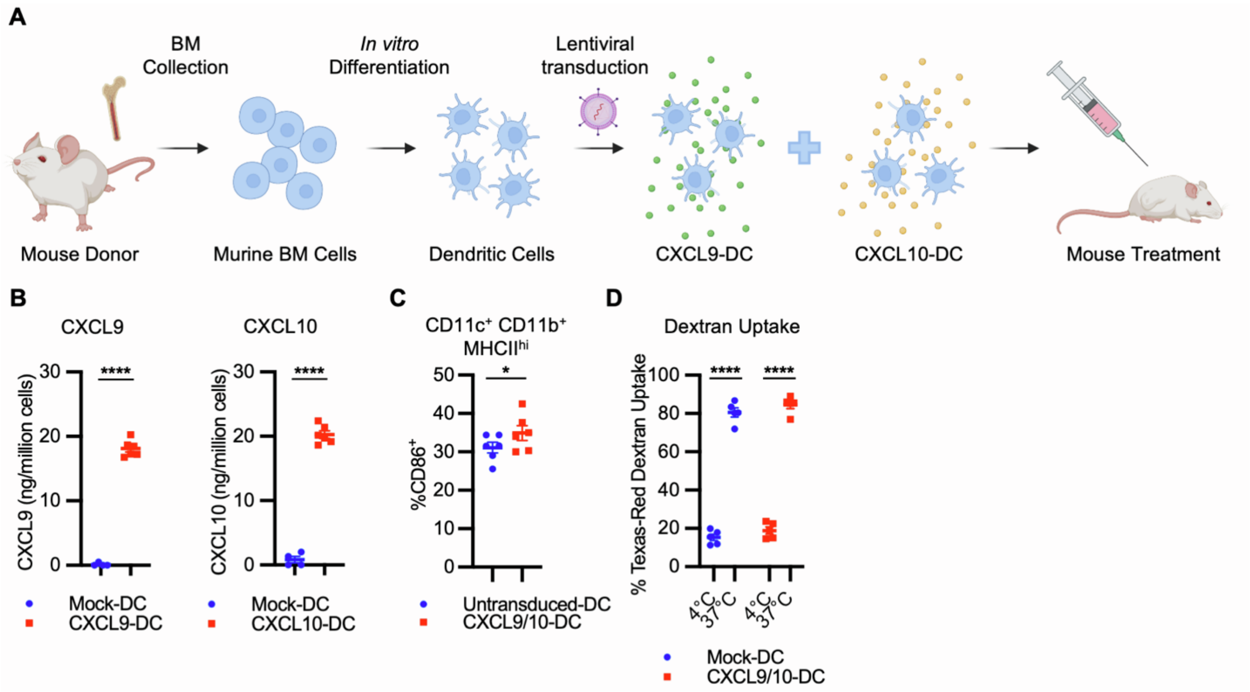

Figure S2. ***In vitro*** **generation of CXCL9/10-DC. Related to Figure 2.**

(**A**) A schematic of *in vitro* generation of CXCL9/10-DC.

(**B**) Validation of CXCL9 and CXCL10 secretion from DC 24 h post lentiviral transduction by ELISA. Vector virus transduced DC (Mock-DC) was included as a control. Each point represents a viral lot (n=6).

(**C**) %$CD86^+$ DC of total DC ($CD11c^+CD11b^+MHCII^{hi}$) with or without lentiviral transduction revealed by flow cytometry (n=6).

(**D**) FITC-Dextran uptake assay. DC phagocytosis was analyzed by FITC-Dextran uptake at 4°C or 37°C and quantified by flow cytometry (n=5).

P values were determined by unpaired *t*-test. *, *P*<0.05; ****, *P*< 0.00005.

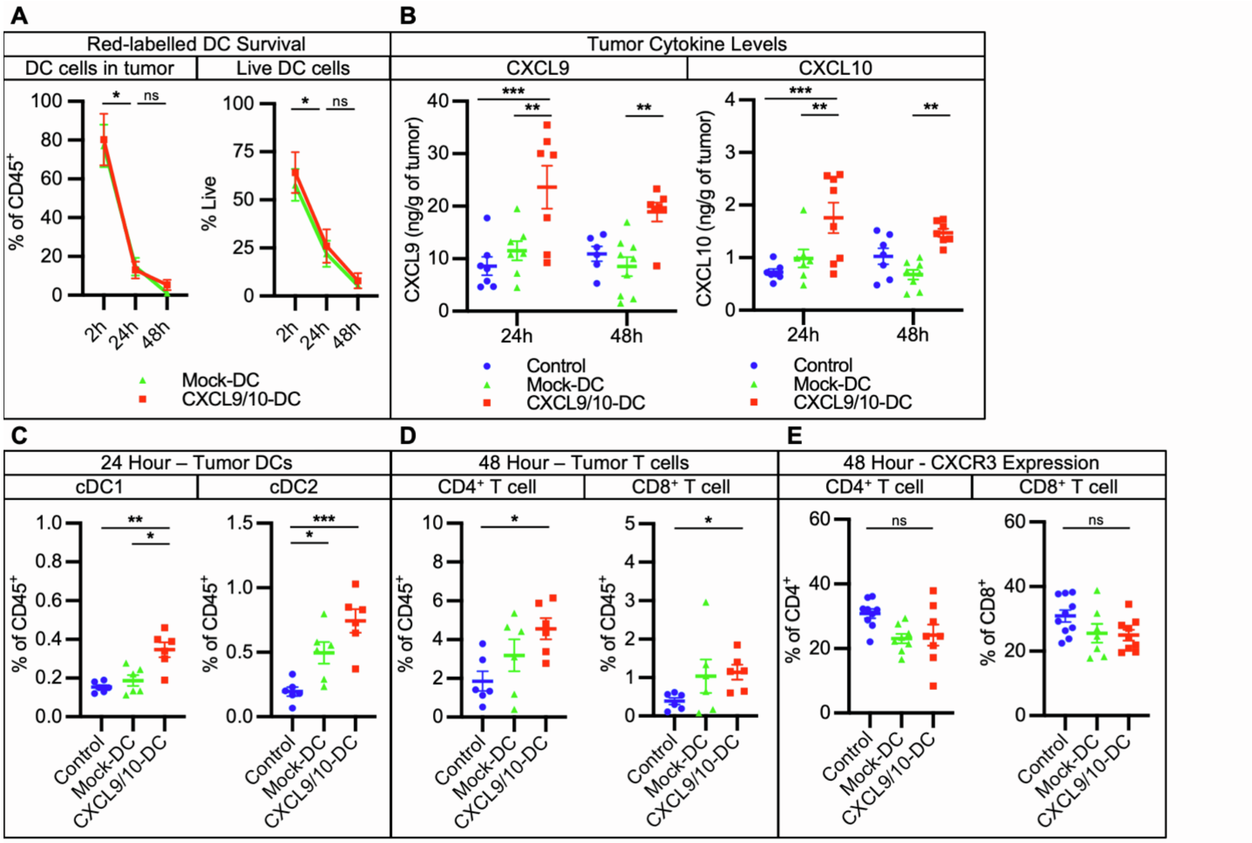

Figure S3. ***In vivo*** **lifespan of DC following IT injections and immediate immune responses. Related to Figure 2.**

FVB mice were inoculated subcutaneously (SC) with 1.5x10^5 KPL-3M cells. On D8 post inoculation, tumors were IT injected with PBS control (blue), 1x10^6 CellTracker Red-labelled mock-DC (green), or CXCL9/10-DC (red) (n=6-8 mice per group). Tumors were harvested at designated timepoints post IT injections for flow cytometry and ELISA.

(**A**) Frequency of Red-labelled DCs in the tumor as a percent of total CD45$^+$ cells and viability of labelled DC by Zombie staining.

(**B**) Levels of CXCL9 and CXCL10 in the tumor at 24h and 48h post IT injections were analyzed by ELISA with total tumor extracts.

(**C**) Frequency of endogenous cDC1s and cDC2s in the tumor as a percent of total CD45$^+$ cells 24h post DC injection.

(**D**) Frequency of endogenous CD4$^+$ and CD8$^+$ T cells in the tumor as a percent of total CD45$^+$ cells 48h post DC injection.

(**E**) CXCR3 expression in tumor infiltrating $CD4^+$ and $CD8^+$ T cells at 48h post IT injections. Error bars represent SEM. *P* values were determined by one-way ANOVA, adjusting for multiple comparisons. ns, not significant; *, P<0.05; **, P< 0.005; ***, P< 0.0005.

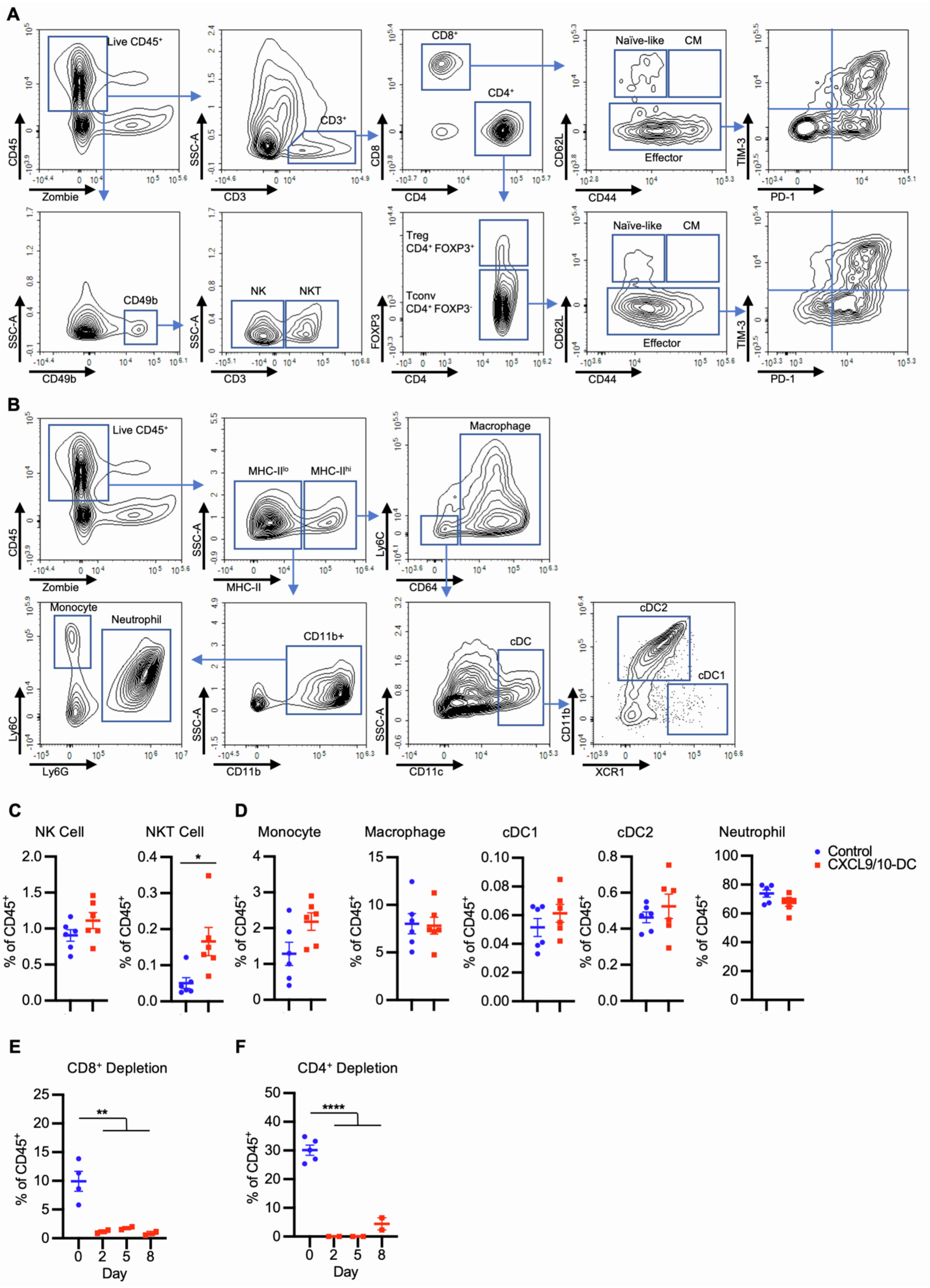
A
Live CD45+
CD45
Zombie
SSC-A
CD3
CD3+
CD8+
CD4+
CD8
CD4
Naïve-like
CM
Effector
CD62L
CD44
TIM-3
PD-1
CD49b
NK
NKT
Treg
CD4+ FOXP3+
Tconv
CD4+ FOXP3-
FOXP3
B
MHC-II
MHC-IIlo
MHC-IIhi
Macrophage
Ly6C
CD64
Monocyte
Neutrophil
Ly6G
CD11b+
CD11b
cDC
CD11c
cDC2
cDC1
XCR1
C
NK Cell
NKT Cell
D
Monocyte
Macrophage
cDC1
cDC2
Neutrophil
% of CD45+
Control
CXCL9/10-DC
E
CD8+ Depletion
F
CD4+ Depletion
Day

Figure S4. Flow phenotyping of immune cell subpopulations following CXCL9/10-DC therapy. Related to Figure 3.

(**A**) Flow cytometry gating strategy of T and NK cells in the tumor.

(**B**) Flow cytometry gating strategy of myeloid cells in the tumor.

(**C**) Tumors were collected on D14 post inoculation (1.25×10^5 KPL-3M delivered SC in FVB mice) following treatment with PBS control or CXCL9/10-DC (IT 1×10^6 CXCL9-DC and CXCL10-DC each/injection on D6, D8 and D11) (n=6-8 mice per group). A significant increase in NKT ($CD45^+CD3^+CD49b^+$) but not NK ($CD45^+CD3^-CD49b^+$) cells was observed in response to CXCL9/10-DC treatment.

(**D**) No significant changes in monocytes ($CD45^+MHCII^{lo}CD11b^+Ly6C^+$), macrophages ($CD45^+MHCII^{hi}CD64^+$), cDC1 ($CD45^+MHCII^{hi}CD11c^+XCR1^+CD11b^-$), cDC2 ($CD45^+MHCII^{hi}CD11c^+$ $XCR1^-CD11b^+$) or neutrophils ($CD45^+MHCII^{lo}CD11b^+Ly6G^+Ly6C^{lo}$) were observed in the same experiment as in **C**.

(**E**) FVB mice were treated with an anti-mouse CD8 depleting antibody (200μg/IP injection) on D0. Mouse blood PBMC was collected on D2, D5 and D8 for flow cytometry measurement of $CD8^+$ T cells to confirm depletion (n=4-5 mice per group).

(**F**) $CD4^+$ T cell depletion was confirmed by flow cytometry as in **E**.

Error bars represent SEM. *P* values were determined by unpaired *t*-test for panels **C-D**, or one-way ANOVA, adjusting for multiple comparisons for panels **E-F**. *, $P<0.05$; **, $P< 0.005$; ****, $P< 0.00005$.

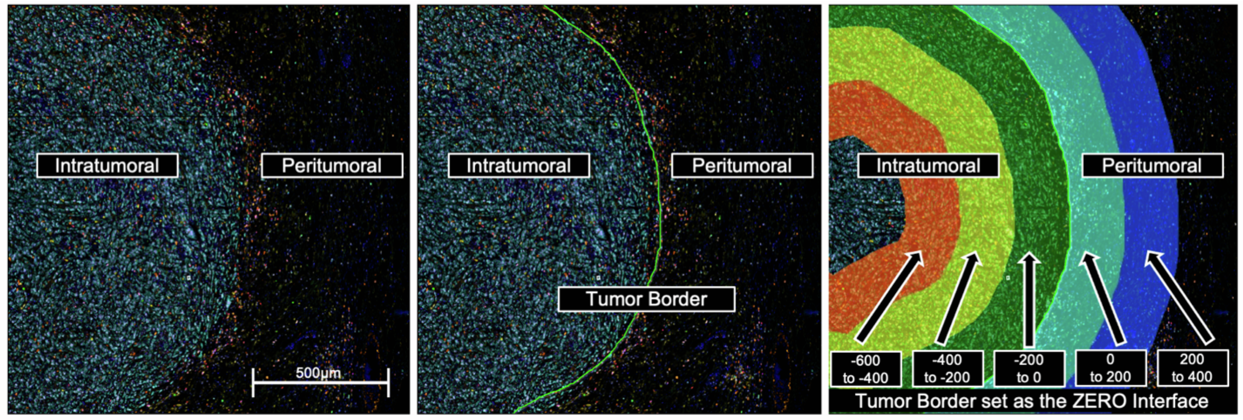

Figure S5. Quantification methods of spatial analysis and T cell recruitment following CXCL9/10-DC. Related to Figure 5.

Tumors were collected on D14 and D23 post inoculation (1.25×10^5 KPL-3M delivered SC in FVB mice) following treatment with PBS control or IT CXCL9/10-DC (1×10^6 CXCL9-DC and CXCL10-DC each/injection on D6, D8 and D11) (n=6-8 mice per group). Tumor tissues were fixed and embedded for MIF staining, followed by cell segmentation and quantification. Spatial infiltration of a given cell type at 200μm intervals was quantified relative to the tumor border (indicated by green line) and corresponding neighboring regions (labeled as intratumoral and peritumoral).

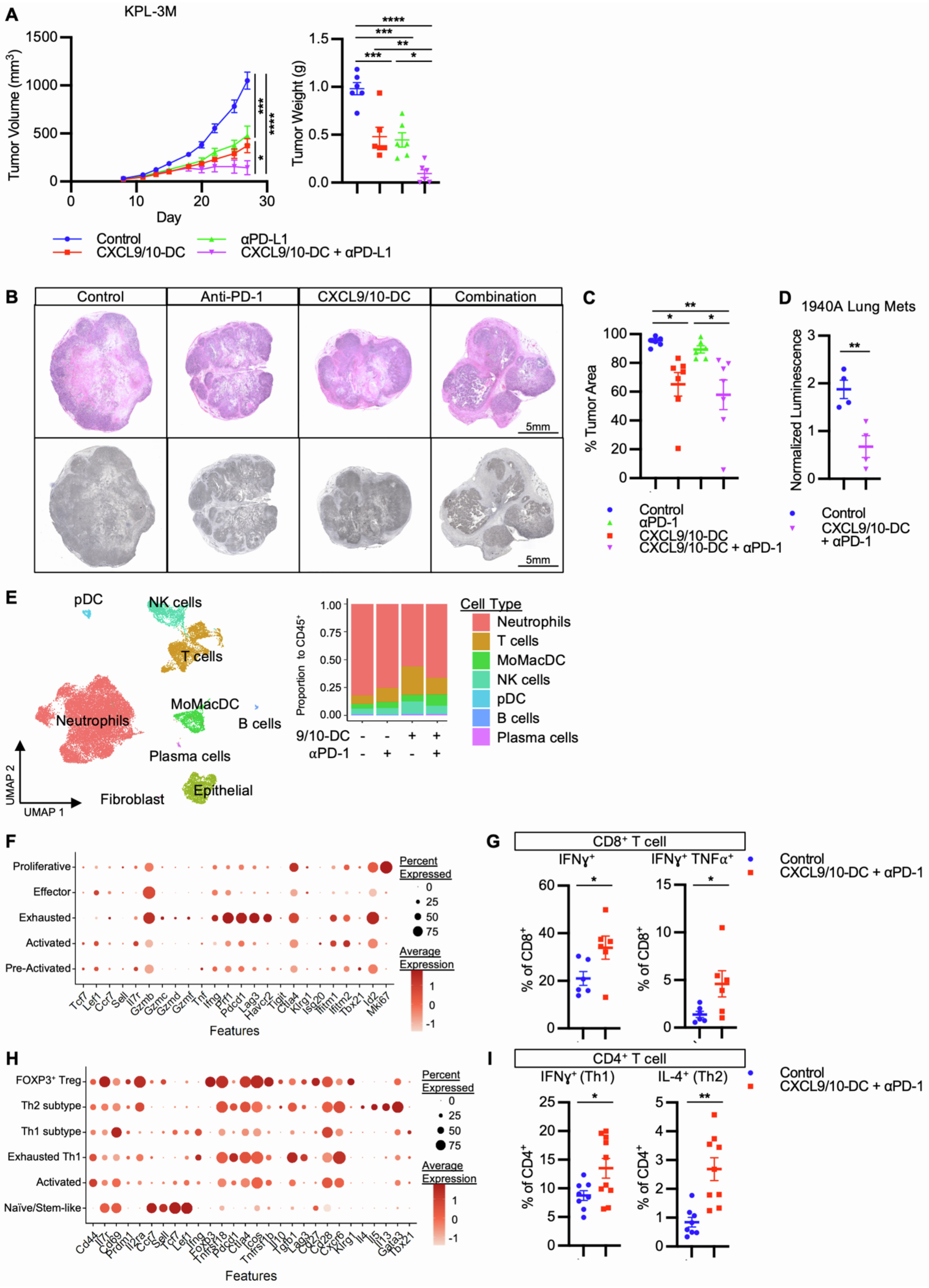
A
KPL-3M
Tumor Volume (mm3)
Day
Tumor Weight (g)
Control
CXCL9/10-DC
αPD-L1
CXCL9/10-DC + αPD-L1
B
Control
Anti-PD-1
CXCL9/10-DC
Combination
5mm
C
% Tumor Area
Control
αPD-1
CXCL9/10-DC
CXCL9/10-DC + αPD-1
D
1940A Lung Mets
Normalized Luminescence
Control
CXCL9/10-DC + αPD-1
E
pDC
NK cells
T cells
Neutrophils
MoMacDC
B cells
Plasma cells
Epithelial
Fibroblast
UMAP 1
UMAP 2
Proportion to CD45+
9/10-DC
αPD-1
Cell Type
Neutrophils
T cells
MoMacDC
NK cells
pDC
B cells
Plasma cells
F
Proliferative
Effector
Exhausted
Activated
Pre-Activated
Tcf7 Lef1 Ccr7 Sell Il7r Gzmb Gzmc Gzmd Gzmf Tnf Ifng Prf1 Pdcd1 Lag3 Havcr2 Tigit Ctla4 Klrg1 Isg20 Ifitm1 Ifitm2 Tbx21 Id2 Mki67
Features
Percent Expressed
Average Expression
G
CD8+ T cell
IFNγ+
IFNγ+ TNFα+
% of CD8+
Control
CXCL9/10-DC + αPD-1
H
FOXP3+ Treg
Th2 subtype
Th1 subtype
Exhausted Th1
Activated
Naïve/Stem-like
Cd44 Il7r Cd69 Prdm1 Il2ra Ccr7 Sell Tcf7 Lef1 Ifng Foxp3 Tnfrsf18 Pdcd1 Ctla4 Icos Tnfrsf1b Il10 Tgfb1 Lag3 Cd27 Cd28 Cxcr6 Klrg1 Il4 Il5 Il13 Gata3 Tbx21
Features
Percent Expressed
Average Expression
I
CD4+ T cell
IFNγ+ (Th1)
IL-4+ (Th2)
% of CD4+
Control
CXCL9/10-DC + αPD-1

Figure S6. Anti-tumor activities of CXCL9/10-DC therapy in combination with ICB, and therapy-induced immune changes in the TME. Related to Figure 6.

(**A**) CXCL9/10-DC enhances the efficacy of anti-PD-L1. On D6 post tumor inoculation ($1.25x10^5$ KPL-3M delivered SC), FVB mice bearing ~$50mm^3$ tumors were randomized and treated with i) PBS control; ii) CXCL9/10-DC (IT $1x10^6$ CXCL9-DC and CXCL10-DC each/injection on D6, D8 and D11); iii) anti-PD-L1 (IP injections at 200 µg/injection on D6, D8, D11 and D14); or iv) the combination of ii) and iii) (n=6-8 mice per group). Tumor growth curves and tumor weights at the time of euthanasia are presented.

(**B**) CXCL9/10-DC treatment reduces tumor density in the KPL-3M model. H&E staining (top) was performed on representative tumors from each treatment group (n=6-8 mice per group) as indicated (top), while single-plex immunofluorescence (bottom) was performed on tumor sections utilizing PanC/K to evaluate tumor density.

(**C**) Quantification of %Tumor area as defined by PanC/K positive regions in relation to total tissue area across the four treatment groups in the KPL-3M model.

(**D**) CXCL9/10-DC and anti-PD-1 combination therapy inhibits lung metastasis in the 1940A-KPL model. Lung tissues from the control and combination treatment groups were harvested at the end of the study in the same experiment as described in **Figure 6B**, digested to single cells, and cultured *in vitro* for 10 days (n=4 per group). Tumor cells were then quantified by a luciferase assay measuring the genetically engineered *Luc* in tumor cells.

(**E**) Therapy-induced changes in $CD45^+$ immune population of each treatment group as measured by single cell RNA sequencing (scRNA-seq) analysis of sorted $CD45^+$ cells (pooled from 5 tumors per group). A total of 31,948 cells passed quality control and clustered into nine major clusters, including epithelial cells, fibroblasts, plasma cells, plasmacytoid DCs (pDC), NK cells, T cells, B cells, neutrophils, and myeloid cells other than neutrophils (MoMacDC).

(**F**) The expression of signature genes in each subcluster of $CD8^+$ T cells revealed by scRNA-seq.

(**G**) Flow cytometry of $CD8^+$ $IFN\gamma^+$ and $CD8^+$ $IFN\gamma^+$ $TNF\alpha^+$ effector T cells following CXCL9/10-DC and anti-PD-1 combination therapy when compared to control at D23 (n=6-8 mice per group).

(**H**) The expression of signature genes in each subcluster of $CD4^+$ T cells revealed by scRNA-seq.

(**I**) Flow cytometry of $CD4^+$ $IFN\gamma^+$ Th1 effector cells and $CD4^+$ IL-4^+ Th2 effector cells following CXCL9/10-DC and anti-PD-1 as in **G**.

Error bars represent SEM. *P* values were determined by one-way ANOVA adjusting for multiple comparisons (**A, C**) or unpaired *t*-test (**D, G, I**). *, $P<0.05$; **, $P< 0.005$; ***, $P< 0.0005$; ****, $P< 0.00005$.

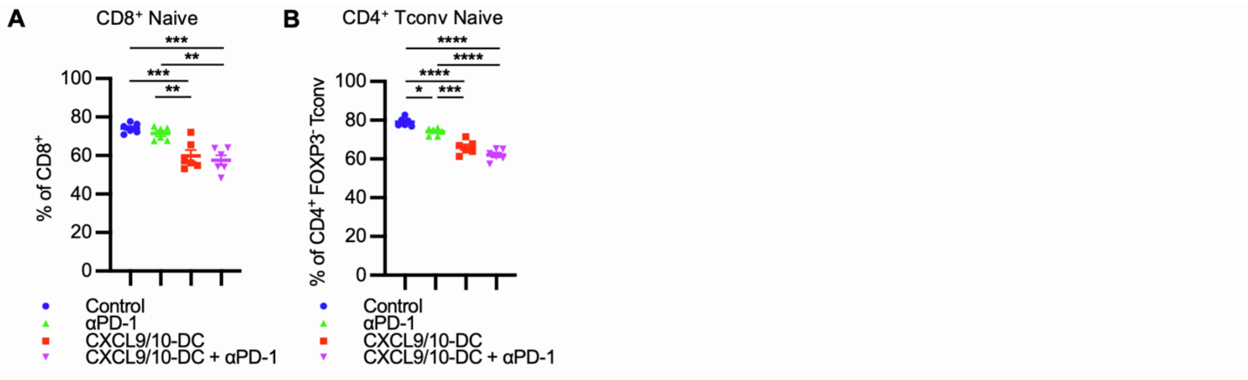

Figure S7. CXCL9/10-DC and anti-PD-1 combination treatment induces systemic immune changes. Related to Figure 7.

Therapy-induced systemic reduction in (**A**) $CD8^+$ or (**B**) $CD4^+$ naïve T cells revealed by flow phenotyping. Spleens were collected on D16 post inoculation ($1.25x10^5$ KPL-3M delivered SC in FVB mice) following treatment with i) PBS control; ii) CXCL9/10-DC (IT $1x10^6$ CXCL9-DC and CXCL10-DC each/injection on D6, D8 and D11); iii) anti-PD-1 (IP injections at 200µg/injection on D6, D8, D11 and D14); or iv) the combination of ii) and iii) (n=6-8 mice per group). Single cell suspensions were prepared and subjected to flow cytometry. Naïve, $CD44^-CD62L^+$. Error bars represent SEM. *P* values were determined by one-way ANOVA adjusting for multiple comparisons. *, *P*<0.05; **, *P*< 0.005; ***, *P*< 0.0005; ****, *P*< 0.00005.
